# Supplementary material for: Associations between hyponatraemia, volume depletion and the risk of falls in US hospitalised patients: a case–control study
Source: BMJ Open. 2017 Aug 7;7(8):e017045. doi: 10.1136/bmjopen-2017-017045 (PMC5724091; doi:10.1136/bmjopen-2017-017045)
Supplement: Author's manuscript [file bmjopen-2017-017045.draft_revisions.pdf]

BMJ Open

BMJ Open is committed to open peer review. As part of this commitment we make the peer review history of every article we publish publicly available.

When an article is published we post the peer reviewers' comments and the authors' responses online. We also post the versions of the paper that were used during peer review. These are the versions that the peer review comments apply to.

The versions of the paper that follow are the versions that were submitted during the peer review process. They are not the versions of record or the final published versions. They should not be cited or distributed as the published version of this manuscript.

BMJ Open is an open access journal and the full, final, typeset and author-corrected version of record of the manuscript is available on our site with no access controls, subscription charges or pay-per-view fees (http://bmjopen.bmj.com).

If you have any questions on BMJ Open's open peer review process please email editorial.bmjopen@bmj.com

Hyponatremia, Volume Depletion, and the Risk of Falls in Hospitalized Patients: A Case-Control Study

Journal:	*BMJ Open*
Manuscript ID	bmjopen-2017-017045
Article Type:	Research
Date Submitted by the Author:	27-Mar-2017
Complete List of Authors:	Fehlberg, Elizabeth; University of Florida, College of Nursing; University of Florida, Clinical and Translational Science Institute<br>Lucero, Robert; University of Florida, College of Nursing; Malcom Randall VA Medical Center, Center for Innovation on Disability and Rehabilitation Research<br>Weaver, Michael; University of Florida, College of Nursing<br>McDaniel, Anna; University of Florida, College of Nursing<br>Chandler, A.; Methodist Healthcare University Hospital<br>Richey, Phyllis; University of Tennessee Health Science Center, Department of Preventative Medicine<br>Mion, Lorraine; The Ohio State University, College of Nursing<br>Shorr, Ronald; Malcom Randall Veterans Affairs Medical Center, Geriatric Research Education and Clinical Center; University of Florida, Epidemiology
**Primary Subject Heading**:	Epidemiology
Secondary Subject Heading:	Nursing, Geriatric medicine
Keywords:	EPIDEMIOLOGY, GERIATRIC MEDICINE, Clinical chemistry < PATHOLOGY, Hospital related, Risk factors, Falls

For peer review only - http://bmjopen.bmj.com/site/about/guidelines.xhtml

Hyponatremia, Volume Depletion, and the Risk of Falls in Hospitalized Patients: A Case-Control Study

Elizabeth A. Fehlberg, BSN, RN [1,2]
Robert J. Lucero, PhD, MPH, RN, FAAN [1,2,3]
Michael T. Weaver PhD, RN, FAAN [1]
Anna M. McDaniel PhD, RN, FAAN [1]
A. Michelle Chandler [4]
Phyllis A. Richey, PhD [5]
Lorraine C. Mion PhD, RN, FAAN [6]
Ronald I. Shorr MD, MS, FACP [2,7,8]

1. University of Florida College of Nursing; Gainesville, FL
2. University of Florida Clinical and Translational Science Institute; Gainesville, FL
3. Center for Innovation on Disability and Rehabilitation Research (CINDRR), Malcom Randall VAMC; Gainesville, FL
4. Methodist Healthcare University Hospital; Memphis, TN
5. Department of Preventive Medicine, University of Tennessee Health Science Center; Memphis, TN
6. College of Nursing, The Ohio State University; Columbus, OH
7. Geriatric Research Education and Clinical Centers (GRECC), Malcom Randall VAMC; Gainesville, FL
8. Department of Epidemiology, University of Florida; Gainesville, FL

Corresponding Author:
Elizabeth A. Fehlberg
Health Professions, Nursing and Pharmacy Complex
1225 Center Drive
PO Box 100197
Gainesville, FL 32610-0197
352-273-6370; Fax: 352-273-6568
efehlberg@ufl.edu

Word Count: 2924

For peer review only

ABSTRACT

OBJECTIVES: Previous research examining volume depletion as a risk factor for hospital-acquired falls has been limited. We aimed to determine if abnormal laboratory values which may indicate volume depletion are associated with increased odds of experiencing a hospital-acquired fall.

DESIGN: Matched case-control.

SETTING: Four hospitals located in the southeast United States.

PARTICIPANTS: Data from 699 adult fallers and 1189 matched controls (non-fallers) were collected via chart review from 2005 to 2010. Controls were matched to cases by nursing unit, time of fall, and length of stay.

OUTCOME MEASURES: The primary exposures included serum sodium, blood urea nitrogen (BUN), creatinine, and BUN/creatinine ratio. Conditional logistic regression with m:n matching was used to determine adjusted and unadjusted odds ratios.

RESULTS: Serum sodium levels were strongly associated with falls. In models controlling for demographic and other fall risk factors, patients with serum sodium levels of 125mEq/L or less were associated with increased odds of experiencing a fall as compared to those with serum sodium levels of greater than 134mEq/L (adjusted odds ratio (aOR) = 5.41, 95% confidence interval (CI)=1.58-18.49). Conversely, elevated BUN, creatinine, and elevated BUN/creatinine ratios were not associated with increased odds of experiencing a fall (aOR=0.63, 95% CI= 0.48-0.82, and aOR=0.69, 95% CI= 0.53-0.90, and aOR=0.77, 95% CI=0.57-1.03, respectively.)

CONCLUSIONS: Volume depletion appears to be unrelated to falls whereas hyponatremia does appear to be a risk factor for falls, and those with serum sodium levels below 126mEq/L are at especially high risk. It may be that other deficits associated with hyponatremia, such as altered

For peer review only

mental status, are associated with risk of experiencing a hospital-acquired fall. These results indicate that abnormal laboratory values, such as low sodium, can be useful for identifying hospitalized patients at risk of falling. Therefore, further investigation into abnormal laboratory values as predictors of hospital-acquired falls is warranted.

Key words: *epidemiology, geriatric medicine, clinical chemistry, hospital related, risk factors, falls*

For peer review only

Strengths and limitations of this study

- The main strengths of this study include: 1) a large sample size collected over six years, 2) the setting which included four hospitals, and 3) a matching strategy that controls for environmental and staffing factors at the time of a fall event.
- Only laboratory values were used as potential indicators of volume depletion instead of other indicators such as urine output or orthostatic blood pressure.
- Our design did not account for all environmental factors such as location of patient room relative to the nursing station.
- Due to the nature of observational research, we cannot exclude the possibility of incomplete control of potential confounders and there were small numbers of patients with serum sodium levels of 125mEq/L or less.
- The generalizability of our findings is limited by the context of our study and the sampling frame which relied on only patients whose laboratory results were known 24 hours prior to the fall index time.

For peer review only

INTRODUCTION

Each year between 700,000 and 1,000,000 falls occur in United States hospitals with associated total direct medical costs of $34 billion[1, 2]. Additionally, falls are the leading cause of both fatal and nonfatal injuries among older adults[1]. Numerous studies have been conducted to examine the associations between patient-level risk factors and hospital-acquired falls. Examples of previously identified risk factors include dementia, diabetes, depression, cognitive status, and impaired mobility[3-9]. Although many researchers have examined demographic factors and health conditions, few studies have aimed to understand the underlying biological mechanisms related to falling. Laboratory values, which have generally been neglected in previous falls research, can potentially increase our understanding of risk factors for hospital-acquired falls[10].

Researchers have created tools to assist in the identification of patients at risk for falls including the Morse Fall Scale, the STRATIFY Scale, and the Hendrich Fall Risk Model[4, 11, 12]. These tools include risk factors such as history of falls, mobility impairments, and altered elimination. However, the predictability of these tools has been shown to vary considerably when subjected to external validations[13]. This indicates that there is potential for improvement which could be achieved with the inclusion of additional factors such as laboratory values. Researchers have examined the diagnosis of anemia, hemoglobin levels, and/or hematocrit levels as potential risk factors for hospital-acquired falls[3, 8, 14-22]. However, there has been limited examination of other laboratory values. Previous research has examined hyponatremia in a psychiatric population, hyponatremia and hypokalemia in a Japanese cohort, albumin in a postoperative population, and creatinine, sodium, and the ratio of blood urea nitrogen (BUN) to creatinine[18, 23-25]. Additionally, one study examined bivariate associations between

For peer review only

approximately thirty laboratory values and falling[17]. However, all of the aforementioned non-anemia laboratory work was limited in sample size, with approximately 100 fallers.

Previous research has shown that volume depletion is a risk factor for falls, and volume depletion can potentially be identified through examination of laboratory values[10, 26, 27]. Abnormal laboratory values which may indicate volume depletion include high BUN levels, high creatinine levels, high BUN to creatinine ratios, or high and low sodium levels[28, 29]. Although previous research has identified volume depletion as a risk factor, to the best of our knowledge no thorough examination of volume depletion and hospital-acquired falls has been performed. We found one study that examined the relationship between fluid intake and falls in a nursing home and found a significant decline in falls (P=0.05) during a hydration program[30]. However, further examination of this potential relationship is warranted, especially since nursing home and hospital populations differ. To address whether volume depletion is potentially related to hospital-acquired falls, we used a case-control design to test if abnormal BUN, creatinine, and sodium levels were associated with odds of a hospital-acquired fall.

For peer review only

METHODS

Conceptual Framework

This study is informed by Choi's conceptual multi-systemic fall prevention model[31]. Intrinsic risk factors, including physiological factors (i.e., laboratory values), are directly associated with fall risk. In addition, symptoms secondary to physiological changes are associated with environmental and technological interventions. By discovering physiological intrinsic risk factors, modifications to the environment and care processes can theoretically lead to a reduction in falls.

Study Population

This case-control study was conducted using data that were collected between 2005 and 2010 across four hospitals. These hospitals are located in the southeastern United States and belong to the same health system. Data from the largest hospital, a 635-bed urban community facility, were collected from fifteen medical-surgical units. Data from the three other suburban community hospitals, ranging in size from 200-260 beds, were collected from nine medical-surgical units. A case was defined as a patient who fell on one of the 24 study units during the study period as reported in the hospital incident reporting system. For each case, we identified up to two controls with a similar hospital length of stay who were patients on the same nursing unit at the time of the fall. We designated an index time for the controls based on the date and time that the matched case fell. The data collection was approved by the Institutional Review Board of the University of Tennessee Health Science Center. This secondary analysis was approved by the University of Florida Institutional Review Board.

Exposures

For peer review only

Exposures were determined for both cases and controls using medical records review by personnel blinded to the case status of the patient. We recorded exposures most proximately preceding the time of fall, or index time, and exposures greater than 24 hours before the event were excluded. Our primary exposures were serum sodium and other abnormal laboratory values that may indicate volume depletion including high BUN, creatinine, and BUN to creatinine ratio. Volume depletion occurs when there is a decrease in circulating blood volume which can occur as a result of inadequate fluid intake or excessive loss of fluids or blood. The abnormal laboratory value classifications can be found in Table 2[28].

Covariates

Demographic covariates in this study included age, race, and gender. We also adjusted for the medical conditions of Parkinson's disease, dementia, hypertension, congestive heart failure (CHF), diabetes mellitus, and stroke. Additionally, we controlled for the patient's fall risk score prior to the fall index time. This score is based on the Morse Fall Scale with the inclusion of medications which potentially effect mobility or cognition (e.g., sedatives, antipsychotics, antidepressants, diuretics, and opiates)[11]. We based the binary measure of high fall risk (Yes/No) on this standardized tool which is used across the four hospitals.

Statistical Analysis

Data analysis was performed using SAS software, Version 9.4 of the SAS System for Windows (SAS Institute, Inc., Cary, NC). Univariate descriptive statistics were calculated by fall status for the overall sample. Descriptive statistics including mean and standard deviation were calculated for continuous data, and counts and percentages were calculated for categorical data. Potential multicollinearity issues were evaluated by testing the correlation between categorical exposures using the phi coefficient. Since cases either had one or two controls, and were

For peer review only

matched based on potential confounders, conditional logistic regression with m:n matching was used to determine bivariate and multivariable associations[32, 33]. Using this approach, the adjusted odds ratios were estimated along with their corresponding 95% confidence intervals. A series of multivariable models were created. Each multivariable model included one of the abnormal laboratory values of interest and the potential covariates. Potential covariates were included in the multivariable models regardless of bivariate *P*-values.

Additional analyses were performed on serum sodium levels to further analyze the potential relationship between hyponatremia and hospital-acquired falls. A categorical variable was created which included three levels of serum sodium including 125mEq/L or lower, 126mEq/L to 134mEq/L, and 135mEq/L or greater. These categories were created to determine if there was a dose-response association between hospital-acquired falls and sodium levels. Conditional logistic regression with m:n matching was used to determine the bivariate and multivariable associations. Using this approach, the unadjusted and adjusted odds ratios were estimated along with their corresponding 95% confidence intervals. A series of models were created including (1) an unadjusted model which only included the categorical sodium level variable (2) an adjusted model which included the categorical sodium level and binary high BUN to creatinine ratio variables, and (3) an adjusted model which included the categorical sodium level variable and the covariates of age, race, gender, high fall risk score, and the medical conditions of dementia, hypertension, CHF, diabetes, and stroke.

For peer review only

RESULTS

The final sample included 699 fallers and 1189 matched controls. Descriptive statistics by fall status for the final sample can be found in Table 1. Controls were 62 years old on average and 59.7% female whereas the cases were 65 years old on average and 50.9% female. Additionally, only 35.8% of controls compared to 55.7% of cases were assessed as high risk prior to the fall index time. Also, 75.4% of cases had a diagnosis of hypertension whereas 68.5% of controls had a diagnosis of hypertension. We did not include the exposures of Parkinson's Disease and high sodium in further analyses due to low frequencies of less than 3% in this sample.

Table 1. Characteristics of cases and controls by Fall Status (N=1888)

	**Controls (n=1189)**			**Cases (n=699)**		
**Factor**	**N**	**Frequency (%)**	**Mean (SD)**	**N**	**Frequency (%)**	**Mean (SD)**
Age	1189		61.6 (17.8)	699		64.9 (16.1)
Race	1189			698		
White		538 (45.3)			392 (56.2)	
Not White		651 (54.8)			306 (43.8)	
Gender	1189			698		
Male		479 (40.3)			343 (49.1)	
Female		710 (59.7)			355 (50.9)	
Hospital	1187			699		
University		528 (44.5)			320 (45.8)	
Community 1		285 (24.0)			164 (23.5)	
Community 2		97 (8.2)			57 (8.2)	
Community 3		277 (23.3)			158 (22.6)	
High fall risk	1169	419 (35.8)		684	381 (55.7)	
Medical conditions						
Parkinson's	1185	12 (1.0)		698	12 (1.7)	
Dementia	1185	139 (11.7)		698	106 (15.2)	
Hypertension	1188	814 (68.5)		699	527 (75.4)	
CHF	1186	252 (21.3)		694	160 (23.1)	
Diabetes	1186	394 (33.2)		696	261 (37.5)	
Stroke	1185	143 (12.1)		695	108 (15.5)	

Note. CHF = Congestive heart failure.

For peer review only

Unadjusted odds ratios from bivariate conditional logistic regression models can be found in Table 2. In the unadjusted model, the presence of low sodium increased the odds of a hospital-acquired fall (odds ratio (OR)=1.485, 95% confidence interval (CI)=1.136-1.940). In contrast, the presence of elevated BUN levels decreased the odds of a hospital-acquired fall (OR=0.785, 95% CI=0.622-0.991). The presence of elevated creatinine levels and BUN to creatinine ratios were not significantly associated with the occurrence of a hospital-acquired fall in the unadjusted models.

Table 2. Bivariate and Multivariable Conditional Logistic Regression of Abnormal Blood Urea Nitrogen (BUN), Creatinine, and Sodium Values on Hospital-Acquired Falls

Model	Factor	Controls (n)	Cases (n)	Unadjusted OR (95% CI)	Adjusted OR[a] (95% CI)
1	BUN > 21mg/dL	337	213	0.785 (0.622, 0.991)	0.627 (0.482, 0.816)
	BUN ≤ 21mg/dL	579	411	Reference	Reference
2	Cr > 1.1mg/dL[b]	363	230	0.827 (0.655, 1.045)	0.692 (0.530, 0.903)
	Cr ≤ 1.1mg/dL[c]	556	397	Reference	Reference
3	BUN/Cr > 20	215	140	0.860 (0.657, 1.124)	0.766 (0.571, 1.028)
	BUN/Cr ≤ 20	700	483	Reference	Reference
4	Na ≥ 135mEq/L	762	469	Reference	Reference
	Na < 135mEq/L	155	148	1.485 (1.136, 1.940)	1.370 (1.025, 1.832)

Note. BUN = Blood Urea Nitrogen, Cr = Creatinine, Na = Sodium.
[a]Conditional logistic regression model adjusted for age, race, gender, high fall risk score, and medical conditions of dementia, hypertension, CHF, diabetes, and stroke
[b]In males > 1.2mg/dL
[c]In males ≤ 1.2mg/dL

Adjusted odds ratios from the four multivariable conditional logistic regression models can also be found in Table 2. Each of these models included one abnormal laboratory value which may indicate volume depletion while controlling for the covariates of age, race, gender, high fall risk score, and the medical conditions of dementia, hypertension, CHF, diabetes, and stroke. After adjusting for these covariates, the presence of low sodium significantly increased the odds of a hospital-acquired fall by 37% (P=0.034). Presence of high BUN and high creatinine were significantly associated with decreased odds of experiencing a hospital-acquired

For peer review only

fall (OR = 0.627, *P*=0.001, and OR=0.692, *P*=0.007, respectively). However, a high BUN to creatinine ratio was not significantly associated with experiencing a hospital-acquired fall in the adjusted model. The results of these four models indicate that based on abnormal laboratory values, volume depletion may not be associated with increased odds of a hospital-acquired fall.

Categories of serum sodium levels including 125mEq/L or lower, 126mEq/L to 134mEq/L, and 135mEq/L or greater were analyzed further. Adjusted and unadjusted odds ratios from conditional logistic regression models for these categories of serum sodium levels can be found in Table 3. Three models were created including an unadjusted model, a model adjusted for presence of a high BUN to creatinine ratio, and a model adjusted for multiple covariates. These covariates included age, race, gender, high fall risk score, and the medical conditions of dementia, hypertension, CHF, diabetes, and stroke. In the unadjusted model, those with a serum sodium level of 125mEq/L or lower were 4.82 times more likely to experience a hospital-acquired fall as compared to a serum sodium level of 135mEq/L or greater (95% CI= 1.54-15.12). In the two models adjusting for presence of a high BUN to creatinine ratio and multiple covariates, those with a serum sodium level of 125mEq/L or lower were 4.80 (95% CI=1.53-15.08) and 5.41 (95% CI=1.58-18.49) times more likely to experience a hospital-acquired fall, respectively.

Table 3. Bivariate and Multivariable Conditional Logistic Regression of Serum Sodium Levels on Hospital-Acquired Falls

**Serum Sodium (mEq/L)**	**Controls (n)**	**Cases (n)**	**Unadjusted OR (95% CI)**	**OR Adjusted for high BUN/Cr (95% CI)**	**OR Adjusted for multiple risk factors[a] (95% CI)**
125 or lower	5	12	4.82 (1.54-15.12)	4.80 (1.53-15.08)	5.41 (1.58-18.49)
126-134	150	136	1.37 (1.04-1.81)	1.39 (1.05-1.83)	1.25 (0.92-1.69)
135 or greater	762	469	Reference	Reference	Reference

[a]Conditional logistic regression model adjusted for age, race, gender, high fall risk score, and medical conditions of dementia, hypertension, CHF, diabetes, and stroke

For peer review only

Additionally, Figure 1 displays the proportion of cases (fallers) and controls (non-fallers) among the three categories of serum sodium levels. Of those with a serum sodium level of 125mEq/L or lower, approximately 71% were fallers and 29% were non-fallers. In contrast, of those with a serum sodium level of 126mEq/L to 134mEq/L and 135mEq/L or greater, approximately 48% and 38% were fallers and 52% and 62% were non-fallers, respectively.

For peer review only

DISCUSSION

In this study, we identified important associations between patient-level risk factors and hospital-acquired falls. Of the four variables chosen as indicators of volume depletion that were usable in the analyses, only low sodium was independently related to increased odds for falling in the hospital setting. However, the abnormal laboratory values of high BUN levels and high creatinine levels were significantly associated with decreased odds of experiencing a hospital-acquired fall. It is possible that high BUN and creatinine levels appear to be protective because high BUN and creatinine levels can result in fatigue[28]. A fatigued patient might ambulate less and therefore be less likely to experience a fall. In addition to potentially indicating volume depletion, electrolyte abnormalities such as low sodium can also result in deficits including altered cognitive status and weakness[10, 28]. Lacking significant positive associations between other volume depletion-related laboratory values, it is likely that the relationship between hyponatremia and hospital-acquired falls is related to deficits arising from hyponatremia rather than directly related to volume depletion. Additionally, we observed that the odds of experiencing a hospital-acquired fall appear to increase as sodium levels decrease independent of a high BUN to creatinine ratio or other risk factors. This pattern further suggests that hyponatremia is a risk factor for experiencing a hospital-acquired fall and that volume depletion does not appear to be the casual pathway.

Our findings add to previous work examining the relationship between hyponatremia and hospital-acquired falls. An observational study conducted in a single hospital in Japan with a sample containing 97 fallers found that when controlling for age, comorbidities, and increases in sedative doses, hyponatremia (serum sodium < 135 mEq/L) significantly increased the odds of experiencing a hospital-acquired fall (OR = 1.751)[25]. Additionally, an observational study

For peer review only

examining hyponatremia and hospital-acquired falls among a psychiatric population found that when controlling for age, antiepileptic drug use, and selective serotonin re-uptake inhibitor (SSRI) use, hyponatremia significantly increased the odds of experiencing a hospital-acquired fall (OR = 4.38)[24]. In contrast, a case-control study found that low sodium was not significantly associated with experiencing a hospital-acquired fall in a population of only those age 65 and older[23]. However, this study was limited by sample size with only 62 fallers and 62 controls which may have limited the statistical power of its findings. Collectively, it is not easy to determine the relative contribution of our work because not all of these studies provided an operational definition of hyponatremia.

The relationship between hyponatremia and non-hospital-acquired falls has also been examined in an observational study of fall-related and non-fall related geriatric trauma admissions. The investigators of that study determined that when controlling for potential confounders such as age and pre-existing conditions, patients with fall-related admissions were significantly more likely to have low sodium levels (OR=1.81)[34]. Also, investigators using a case-control study design including patients admitted to the emergency room with and without chronic hyponatremia, found that patients with chronic hyponatremia were significantly more likely to have experienced a fall after controlling for covariates (OR=67)[35]. In a similar case-control study including geriatric patients that were admitted with and without hyponatremia, investigators found that when controlling for covariates such as age, gender, admitting diagnosis, and medications, patients with hyponatremia were significantly more likely to have a fall associated with their admission (e.g., as a presenting complaint) (OR=3.12)[36]. Additionally, investigators examining hyponatremia among community-dwelling older adults found that after

For peer review only

adjusting for age, gender, and diuretic use, persons with hyponatremia were significantly more likely to have experienced a fall (P=0.01)[37].

Unlike most previous work related to laboratory values and falls, our study is informed by a conceptual framework that hypothesized the relationship between laboratory values and fall risk[31]. Additional strengths of this study include the matching strategy and large sample size. Matching was based on nursing unit, date and time of fall, and length of stay which should control for environmental and staffing factors at the time of the fall. Thus, this matching strategy is best for identifying patient-level fall risk factors. In previous research, measures of the hospital environment such as physical measures (e.g., unit layout), resource availability measures (e.g., nurse staffing), and culture measures (e.g., magnet or teaching status) have been significantly associated with patient outcomes[38-40]. Additionally, at the unit environment level, researchers have found that some medical units tend to have persistently low fall rates whereas other medical units have persistently high fall rates[40]. The environment appears to have an effect on patient falls and it is therefore important to account for this variation. However, it should also be noted that our design cannot control for all environmental factors such as location of patient room relative to the nursing station.

A limitation of this study is that only laboratory values were used as potential indicators of volume depletion. Future work to further examine the potential relationship between volume depletion and hospital-acquired falls should consider using other potential indicators such as urine output, urine specific gravity or orthostatic blood pressure. However, laboratory values are frequently collected in the hospital setting and are relatively reliable measures that have been neglected in previous falls research. Also, this study is limited by selecting for only those hospitalized patients who received laboratory results within their last 24 hours of hospitalization

For peer review only

prior to the fall index time. This, as well as sampling from one area of the country, limits the generalizability of these findings. However, the sample for this study was collected from four hospitals which contributes to the generalizability of these findings. Additional limitations include that observational research is susceptible to threats to internal validity including the incomplete control of potential confounders. However, we believe that our matching strategy helped to control for the relevant potential confounders of length of stay, nurse staffing, unit culture, and unit environment.

CONCLUSION

In this matched case-control study, we found a strong relationship between hyponatremia and fall risk in hospitalized patients. This relationship is independent of an increased BUN to creatinine ratio, demographic risk factors, and other patient-level risk factors for hospital falls. Conversely, we found no other associations of laboratory findings consistent with volume depletion and increased risk of falls. It is possible that other indicators of volume depletion, such as orthostatic hypotension, are necessary for the examination of this relationship. The results of this study do indicate that abnormal laboratory values, such as low sodium, can be useful for identifying hospitalized patients at increased risk of experiencing a fall. Symptoms associated with hyponatremia, including mental status changes, can be addressed with system- and patient-level interventions such as modifying the patient environment and regular patient surveillance. Further investigation into abnormal laboratory values as predictors of hospitalized-acquired falls is warranted, and if validated, should be added to currently utilized fall risk scales.

For peer review only

Funding:
This work was supported by the National Institute on Aging (R01-AG033005 to R.I.S.); and the National Center for Advancing Translational Sciences of the National Institutes of Health under University of Florida Clinical and Translational Science Awards (TL1TR001428 and UL1TR001427 to E.A.F.).

The content is solely the responsibility of the authors and does not necessarily represent the official views of the National Institutes of Health.

Competing Interests:
All authors have completed the ICMJE uniform disclosure form at www.icmje.org/coi_disclosure.pdf and declare: no support from any organization for the submitted work other than government funding which is specified in the Funding statement; no financial relationships with any organizations that might have an interest in the submitted work in the previous three years; no other relationships or activities that could appear to have influenced the submitted work except that RIS serves as an expert witness in cases of hospital falls.

Author Contributions:
All authors were involved in formulating the study concept/design. AMC, PAR, and RIS were involved in the acquisition of the data and EAF, MTW, and RIS performed the statistical analysis. EAF, RJL, MTW, AMM, LCM, and RIS were involved in the interpretation of the data, and all of the authors participated in the preparation of this manuscript. Additionally, all authors approved of the submitted manuscript.

For peer review only

REFERENCES

1. Centers for Disease Control and Prevention. Falls among older adults: An overview. https://www.cdc.gov/HomeandRecreationalSafety/Falls/adultfalls.html (Accessed June 2015).

2. Ganz DA, Huang C, Saliba D, et al. Preventing falls in hospitals: a toolkit for improving quality of care. (Prepared by RAND Corporation, Boston University School of Public Health, and ECRI Institute under Contract No. HHSA290201000017I TO #1.). Rockville, MD: Agency for Healthcare Research and Quality 2013. https://www.ahrq.gov/sites/default/files/publications/files/fallpxtoolkit.pdf (Accessed June 2015).

3. Angalakuditi M, Coley KC, Kirisci L, et al. A case-control study to assess the impact of anemia and other risk factors for in-hospital falls. *J Patient Saf* 2007;3(1):16-21. doi: 10.1097/pts.0b013e318030c2ff

4. Hendrich A, Nyhuis A, Kippenbrock T, et al. Hospital falls: development of a predictive model for clinical practice. *Appl Nurs Res* 1995;8(3):129-39.

5. Hendrich AL, Bender PS, Nyhuis A. Validation of the Hendrich II Fall Risk Model: a large concurrent case/control study of hospitalized patients. *Appl Nurs Res* 2003;16(1):9-21. doi: 10.1053/apnr.2003.YAPNR2

6. Krauss MJ, Evanoff B, Hitcho E, et al. A case-control study of patient, medication, and care-related risk factors for inpatient falls. *J Gen Intern Med* 2005;20(2):116-22. doi: 10.1111/j.1525-1497.2005.40171.x

7. Yauk S, Hopkins BA, Phillips CD, et al. Predicting in-hospital falls: development of the Scott and White Falls Risk Screener. *J Nurs Care Qual* 2005;20(2):128-33.

For peer review only

8. Morse JM, Tylko SJ, Dixon HA. Characteristics of the fall-prone patient. *Gerontologist* 1987;27(4):516-22.

9. Ash KL, MacLeod P, Clark L. A case control study of falls in the hospital setting. *J Gerontol Nurs* 1998;24(12):7-15.

10. Rao SS. Prevention of falls in older patients. *Am Fam Physician* 2005;72(1):81-8.

11. Morse JM, Morse RM, Tylko SJ. Development of a scale to identify the fall-prone patient. *Can J Aging* 1989;8(4):366-77. doi: 10.1017/s0714980800008576

12. Oliver D, Britton M, Seed P, et al. Development and evaluation of evidence based risk assessment tool (STRATIFY) to predict which elderly inpatients will fall: case-control and cohort studies. *BMJ* 1997;315(7115):1049-53.

13. Aranda-Gallardo M, Morales-Asencio JM, Canca-Sanchez JC, et al. Instruments for assessing the risk of falls in acute hospitalized patients: a systematic review and meta-analysis. *BMC Health Serv Res* 2013;13(1):122. doi: 10.1186/1472-6963-13-122

14. Chen YC, Chien SF, Chen LK. Risk factors associated with falls among Chinese hospital inpatients in Taiwan. *Arch Gerontol Geriatr* 2009;48(2):132-6. doi: 10.1016/j.archger.2007.11.006

15. Plati C, Lanara V, Mantas J. Risk factors responsible for patients' falls. *Scand J Caring Sci* 1992;6(2):113-8.

16. Dharmarajan TS, Avula S, Norkus EP. Anemia increases risk for falls in hospitalized older adults: an evaluation of falls in 362 hospitalized, ambulatory, long-term care, and community patients. *J Am Med Dir Assoc* 2006;7(5):287-93. doi: 10.1016/j.jamda.2005.10.010

For peer review only

17. O'Hagan C, O'Connell B. The relationship between patient blood pathology values and patient falls in an acute-care setting: a retrospective analysis. *Int J Nurs Pract* 2005;11(4):161-8. doi: 10.1111/j.1440-172X.2005.00519.x

18. Church S, Robinson TN, Angles EM, et al. Postoperative falls in the acute hospital setting: characteristics, risk factors, and outcomes in males. *Am J Surg* 2011;201(2):197-202. doi: 10.1016/j.amjsurg.2009.12.013

19. Capone LJ, Albert NM, Bena JF, et al. Predictors of a fall event in hospitalized patients with cancer. *Oncol Nurs Forum* 2012;39(5):E407-15. doi: 10.1188/12.onf.e407-e415

20. Byers V, Arrington ME, Finstuen K. Predictive risk factors associated with stroke patient falls in acute care settings. *J Neurosci Nurs* 1990;22(3):147-54.

21. Stenvall M, Olofsson B, Lundstrom M, et al. Inpatient falls and injuries in older patients treated for femoral neck fracture. *Arch Gerontol Geriatr* 2006;43(3):389-99. doi: 10.1016/j.archger.2006.01.004

22. Chu LW, Pei CK, Chiu A, et al. Risk factors for falls in hospitalized older medical patients. *J Gerontol A Biol Sci Med Sci* 1999;54(1):M38-43.

23. Walker PC, Alrawi A, Mitchell JF, et al. Medication use as a risk factor for falls among hospitalized elderly patients. *Am J Health Syst Pharm* 2005;62(23):2495-9. doi: 10.2146/ajhp050116

24. Bun S, Serby MJ, Friedmann P. Psychotropic medication use, hyponatremia, and falls in an inpatient population: a retrospective study. *J Clin Psychopharmacol* 2011;31(3):395-7.

25. Tachi T, Yokoi T, Goto C, et al. Hyponatremia and hypokalemia as risk factors for falls. *Eur J Clin Nutr* 2015;69(2):205-10. doi: 10.1038/ejcn.2014.195

For peer review only

26. Rubenstein LZ. Falls in older people: epidemiology, risk factors and strategies for prevention. *Age and Ageing* 2006;35:37-41. doi: 10.1093/ageing/afl084

27. Ungar A, Rafanelli M, Iacomelli I, et al. Fall prevention in the elderly. *Clin Cases Miner Bone Metab* 2013;10(2):91-5.

28. Van Leeuwen AM, Bladh ML. Davis's Comprehensive Handbook of Laboratory & Diagnostic Tests with Nursing Implications. 6th ed. Philadelphia, PA: F.A. Davis Co. 2015.

29. Simon EE. Hyponatremia Evaluation and Treatment. New York, NY: Springer 2013.

30. Robinson SB, Rosher RB. Can a beverage cart help improve hydration? *Geriatr Nurs* 2002;23(4):208-11. doi: 10.1067/mgn.2002.126967

31. Choi YS, Lawler E, Boenecke CA, et al. Developing a multi-systemic fall prevention model, incorporating the physical environment, the care process and technology: a systematic review. *J Adv Nurs* 2011;67(12):2501-24. doi: 10.1111/j.1365-2648.2011.05672.x

32. SAS Institute Inc. SAS/STAT 9.2 User's Guide. 2nd ed., Conditional logisitc regression for matched pairs data. Cary, NC: SAS Institute Inc. 2009.

33. Allison PD. Logistic Regression Using SAS: Theory and Application. 2nd ed. Cary, NC: SAS Institute Inc. 2012. https://support.sas.com/documentation/cdl/en/statug/63033/HTML/default/viewer.htm#statug_logistic_sect062.htm (Accessed August 2016).

34. Rittenhouse KJ, To T, Rogers A, et al. Hyponatremia as a fall predictor in a geriatric trauma population. *Injury* 2015;46(1):119-23. doi: 10.1016/j.injury.2014.06.013

For peer review only

35. Renneboog B, Musch W, Vandemergel X, et al. Mild chronic hyponatremia is associated with falls, unsteadiness, and attention deficits. *Am J Med* 2006;119(1):71.e1-8. doi: 10.1016/j.amjmed.2005.09.026

36. Ahamed S, Anpalahan M, Savvas S, et al. Hyponatraemia in older medical patients: implications for falls and adverse outcomes of hospitalisation. *Intern Med J* 2014;44(10):991-97. doi: 10.1111/imj.12535

37. Gunathilake R, Oldmeadow C, McEvoy M, et al. Mild hyponatremia is associated with impaired cognition and falls in community-dwelling older persons. *J Am Geriatr Soc* 2013;61(10):1838-9. doi: 10.1111/jgs.12468

38. Lake ET, Shang J, Klaus S, et al. Patient falls: Association with hospital Magnet status and nursing unit staffing. *Res Nurs Health* 2010;33(5):413-25. doi: 10.1002/nur.20399

39. Everhart D, Schumacher JR, Duncan RP, et al. Determinants of hospital fall rate trajectory groups: a longitudinal assessment of nurse staffing and organizational characteristics. *Health Care Manage Rev* 2014;39(4):352-60. doi: 10.1097/HMR.0000000000000013

40. Staggs VS, Mion LC, Shorr RI. Consistent differences in medical unit fall rates: implications for research and practice. *J Am Geriatr Soc* 2015;63(5):983-7. doi: 10.1111/jgs.13387

For peer review only

CAPTIONS

Figure 1. Proportions of Cases and Controls Among Serum Sodium Levels.

This figure displays three categories of serum sodium levels including 125mEq/L or lower, 126mEq/L to 134mEq/L, and 135mEq/L or greater. Of those cases and controls with these three different serum sodium levels, the black bars indicate the proportion that are cases and the grey bars indicate the proportion that are controls. Included at the bottom of the figure, is the number of cases and controls within each category of serum sodium level.

For peer review only

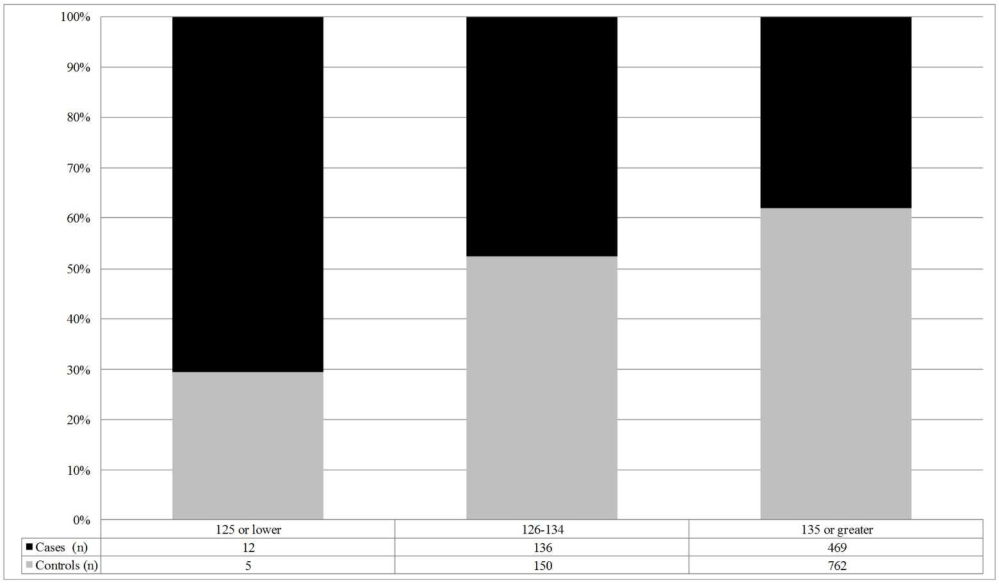

Figure 1. Proportions of Cases and Controls Among Serum Sodium Levels.
This figure displays three categories of serum sodium levels including 125mEq/L or lower, 126mEq/L to 134mEq/L, and 135mEq/L or greater. Of those cases and controls with these three different serum sodium levels, the black bars indicate the proportion that are cases and the grey bars indicate the proportion that are controls. Included at the bottom of the figure, is the number of cases and controls within each category of serum sodium level.

239x139mm (300 x 300 DPI)

STROBE 2007 (v4) Statement—Checklist of items that should be included in reports of *case-control studies*

Section/Topic	Item #	Recommendation	Reported on page #
**Title and abstract**	1	(*a*) Indicate the study's design with a commonly used term in the title or the abstract	1-2
		(*b*) Provide in the abstract an informative and balanced summary of what was done and what was found	2
**Introduction**			
Background/rationale	2	Explain the scientific background and rationale for the investigation being reported	5-6
Objectives	3	State specific objectives, including any prespecified hypotheses	6
**Methods**			
Study design	4	Present key elements of study design early in the paper	7-8
Setting	5	Describe the setting, locations, and relevant dates, including periods of recruitment, exposure, follow-up, and data collection	7
Participants	6	(*a*) Give the eligibility criteria, and the sources and methods of case ascertainment and control selection. Give the rationale for the choice of cases and controls	7
		(*b*) For matched studies, give matching criteria and the number of controls per case	7
Variables	7	Clearly define all outcomes, exposures, predictors, potential confounders, and effect modifiers. Give diagnostic criteria, if applicable	7-8, 11
Data sources/ measurement	8*	For each variable of interest, give sources of data and details of methods of assessment (measurement). Describe comparability of assessment methods if there is more than one group	7-8, 11
Bias	9	Describe any efforts to address potential sources of bias	7
Study size	10	Explain how the study size was arrived at	n/a, based on number of falls occurring during study period-pg7
Quantitative variables	11	Explain how quantitative variables were handled in the analyses. If applicable, describe which groupings were chosen and why	8-9
Statistical methods	12	(*a*) Describe all statistical methods, including those used to control for confounding	8-9
		(*b*) Describe any methods used to examine subgroups and interactions	n/a
		(*c*) Explain how missing data were addressed	n/a, large sample size

For peer review only

		(*d*) If applicable, explain how matching of cases and controls was addressed	8
		(*e*) Describe any sensitivity analyses	n/a
**Results**			
Participants	13*	(a) Report numbers of individuals at each stage of study—eg numbers potentially eligible, examined for eligibility, confirmed eligible, included in the study, completing follow-up, and analysed	10
		(b) Give reasons for non-participation at each stage	n/a, secondary analysis
		(c) Consider use of a flow diagram	n/a
Descriptive data	14*	(a) Give characteristics of study participants (eg demographic, clinical, social) and information on exposures and potential confounders	10-11
		(b) Indicate number of participants with missing data for each variable of interest	10-11
Outcome data	15*	Report numbers in each exposure category, or summary measures of exposure	10-12
Main results	16	(*a*) Give unadjusted estimates and, if applicable, confounder-adjusted estimates and their precision (eg, 95% confidence interval). Make clear which confounders were adjusted for and why they were included	11-12
		(*b*) Report category boundaries when continuous variables were categorized	11-12
		(*c*) If relevant, consider translating estimates of relative risk into absolute risk for a meaningful time period	n/a
Other analyses	17	Report other analyses done—eg analyses of subgroups and interactions, and sensitivity analyses	n/a
**Discussion**			
Key results	18	Summarise key results with reference to study objectives	14
Limitations	19	Discuss limitations of the study, taking into account sources of potential bias or imprecision. Discuss both direction and magnitude of any potential bias	16-17
Interpretation	20	Give a cautious overall interpretation of results considering objectives, limitations, multiplicity of analyses, results from similar studies, and other relevant evidence	14-17
Generalisability	21	Discuss the generalisability (external validity) of the study results	16-17
**Other information**			
Funding	22	Give the source of funding and the role of the funders for the present study and, if applicable, for the original study on which the present article is based	18

For peer review only

BMJ Open

Associations Between Hyponatremia, Volume Depletion, and the Risk of Falls in Hospitalized Patients: A Case-Control Study

Journal:	*BMJ Open*
Manuscript ID	bmjopen-2017-017045.R1
Article Type:	Research
Date Submitted by the Author:	09-May-2017
Complete List of Authors:	Fehlberg, Elizabeth; University of Florida, College of Nursing; University of Florida, Clinical and Translational Science Institute<br>Lucero, Robert; University of Florida, College of Nursing; Malcom Randall VA Medical Center, Center for Innovation on Disability and Rehabilitation Research<br>Weaver, Michael; University of Florida, College of Nursing<br>McDaniel, Anna; University of Florida, College of Nursing<br>Chandler, A.; Methodist Healthcare University Hospital<br>Richey, Phyllis; University of Tennessee Health Science Center, Department of Preventative Medicine<br>Mion, Lorraine; The Ohio State University, College of Nursing<br>Shorr, Ronald; Malcom Randall Veterans Affairs Medical Center, Geriatric Research Education and Clinical Center; University of Florida, Epidemiology
**Primary Subject Heading**:	Epidemiology
Secondary Subject Heading:	Nursing, Geriatric medicine
Keywords:	EPIDEMIOLOGY, GERIATRIC MEDICINE, Clinical chemistry < PATHOLOGY, Hospital related, Risk factors, Falls

SCHOLARONE™ Manuscripts

Associations Between Hyponatremia, Volume Depletion, and the Risk of Falls in Hospitalized Patients: A Case-Control Study

Elizabeth A. Fehlberg, BSN, RN [1,2]
Robert J. Lucero, PhD, MPH, RN, FAAN [1,2,3]
Michael T. Weaver PhD, RN, FAAN [1]
Anna M. McDaniel PhD, RN, FAAN [1]
A. Michelle Chandler [4]
Phyllis A. Richey, PhD [5]
Lorraine C. Mion PhD, RN, FAAN [6]
Ronald I. Shorr MD, MS, FACP [2,7,8]

1. University of Florida College of Nursing; Gainesville, FL
2. University of Florida Clinical and Translational Science Institute; Gainesville, FL
3. Center for Innovation on Disability and Rehabilitation Research (CINDRR), Malcom Randall VAMC; Gainesville, FL
4. Methodist Healthcare University Hospital; Memphis, TN
5. Department of Preventive Medicine, University of Tennessee Health Science Center; Memphis, TN
6. College of Nursing, The Ohio State University; Columbus, OH
7. Geriatric Research Education and Clinical Centers (GRECC), Malcom Randall VAMC; Gainesville, FL
8. Department of Epidemiology, University of Florida; Gainesville, FL

Corresponding Author:
Elizabeth A. Fehlberg
Health Professions, Nursing and Pharmacy Complex
1225 Center Drive
PO Box 100197
Gainesville, FL 32610-0197
352-273-6370; Fax: 352-273-6568
efehlberg@ufl.edu

Word Count: 3270

For peer review only

ABSTRACT

OBJECTIVES: Previous research examining volume depletion as a risk factor for hospital-acquired falls has been limited. We aimed to determine if abnormal laboratory values which may indicate volume depletion are associated with increased odds of experiencing a hospital-acquired fall.

DESIGN: Matched case-control.

SETTING: Four hospitals located in the southeast United States.

PARTICIPANTS: Data from 699 adult fallers and 1189 matched controls (non-fallers) were collected via chart review from 2005 to 2010. Controls were matched to cases by nursing unit, time of fall, and length of stay.

OUTCOME MEASURES: The primary exposures included serum sodium, blood urea nitrogen (BUN), creatinine, BUN/creatinine ratio, and hematocrit. Conditional logistic regression with m:n matching was used to determine adjusted and unadjusted odds ratios.

RESULTS: Serum sodium levels were strongly associated with falls. In models controlling for demographic and other fall risk factors, patients with serum sodium levels of 125mEq/L or less were associated with increased odds of experiencing a fall as compared to those with serum sodium levels of greater than 134mEq/L (adjusted odds ratio (aOR) = 5.08, 95% confidence interval (CI)=1.43-18.08). Conversely, elevated BUN, creatinine, and elevated BUN/creatinine ratios were not associated with increased odds of experiencing a fall (aOR=0.64, 95% CI=0.49-0.84, and aOR=0.70, 95% CI=0.54-0.92, and aOR=0.77, 95% CI=0.58-1.04, respectively.)

CONCLUSIONS: Volume depletion appears to be unrelated to falls whereas hyponatremia does appear to be a risk factor for falls, and those with serum sodium levels below 126mEq/L are at especially high risk. It may be that other deficits associated with hyponatremia, such as altered

For peer review only

mental status, are associated with risk of experiencing a hospital-acquired fall. These results indicate that abnormal laboratory values, such as low sodium, can be useful for identifying hospitalized patients at risk of falling. Therefore, further investigation into abnormal laboratory values as predictors of hospital-acquired falls is warranted.

Key words: *epidemiology, geriatric medicine, clinical chemistry, hospital related, risk factors, falls*

For peer review only

Strengths and limitations of this study

- The main strengths of this study include: 1) a large sample size collected over six years, 2) the setting which included four hospitals, and 3) a matching strategy that controls for environmental and staffing factors at the time of a fall event.
- Only laboratory values that are not specific for volume depletion were used as potential indicators of volume depletion instead of other indicators such as urine output or orthostatic blood pressure.
- Our design did not account for all environmental factors such as location of patient room relative to the nursing station.
- Due to the nature of observational research, we cannot exclude the possibility of incomplete control of potential confounders and there were small numbers of patients with serum sodium levels of 125mEq/L or less.
- The generalizability of our findings is limited by the context of our study and the sampling frame which relied on only patients whose laboratory results were known 24 hours prior to the fall index time.

For peer review only

INTRODUCTION

Each year between 700,000 and 1,000,000 falls occur in United States hospitals with associated total direct medical costs of $34 billion[1, 2]. Additionally, falls are the leading cause of both fatal and nonfatal injuries among older adults[1]. Numerous studies have been conducted to examine the associations between patient-level risk factors and hospital-acquired falls. Examples of previously identified risk factors include dementia, diabetes, depression, cognitive status, and impaired mobility[3-9]. Although many researchers have examined demographic factors and health conditions, few studies have aimed to understand the underlying biological mechanisms related to falling. Laboratory values, which have generally been neglected in previous falls research, can potentially increase our understanding of risk factors for hospital-acquired falls[10].

Researchers have created tools to assist in the identification of patients at risk for falls including the Morse Fall Scale, the STRATIFY Scale, and the Hendrich Fall Risk Model[4, 11, 12]. These tools include risk factors such as history of falls, mobility impairments, and altered elimination. However, the predictability of these tools has been shown to vary considerably when subjected to external validations[13]. This indicates that there is potential for improvement which could be achieved with the inclusion of additional factors such as laboratory values. Researchers have examined the diagnosis of anemia, hemoglobin levels, and/or hematocrit levels as potential risk factors for hospital-acquired falls[3, 8, 14-22]. However, there has been limited examination of other laboratory values. Previous research has examined hyponatremia in a psychiatric population, hyponatremia and hypokalemia in a Japanese cohort, albumin in a postoperative population, and creatinine, sodium, and the ratio of blood urea nitrogen (BUN) to creatinine[18, 23-25]. Additionally, one study examined bivariate associations between

For peer review only

approximately thirty laboratory values and falling[17]. However, all of the aforementioned non-anemia laboratory work was limited in sample size, with approximately 100 fallers.

Previous research has identified volume depletion as a risk factor for falling, but to the best of our knowledge there has been limited examination of volume depletion and hospital-acquired falls. We found one study that examined the relationship between fluid intake and falls in a nursing home and found a significant decline in falls (P=0.05) during a hydration program[26]. However, further examination of this potential relationship is warranted, especially since nursing home and hospital populations differ. Abnormal serum creatinine, BUN, sodium, and hematocrit levels can indicate multiple health conditions, including volume depletion[10, 27-35]. Specifically, abnormal laboratory values which may indicate volume depletion include high BUN levels (>21mg/dL), high creatinine levels (>1.1mg/dL in females, >1.2mg/dL in males), high BUN to creatinine ratios (>20), high hematocrit levels (>45% in females and >51% in males), or high and low sodium levels (>145mEq/L and <135mEq/L, respectively)[36, 37]. To address whether volume depletion is potentially related to hospital-acquired falls, we used a case-control design to test if abnormal BUN, creatinine, hematocrit, and sodium levels were associated with odds of a hospital-acquired fall.

For peer review only

METHODS

Conceptual Framework

This study is informed by Choi's conceptual multi-systemic fall prevention model[38]. Intrinsic risk factors, including physiological factors (i.e., laboratory values), are directly associated with fall risk. In addition, symptoms secondary to physiological changes are associated with environmental and technological interventions. By discovering physiological intrinsic risk factors, modifications to the environment and care processes can theoretically lead to a reduction in falls.

Study Population

This case-control study was conducted using data that were collected between 2005 and 2010 across four hospitals. These hospitals are located in the southeastern United States and belong to the same health system. Data from the largest hospital, a 635-bed urban community facility, were collected from fifteen medical-surgical units. Data from the three other suburban community hospitals, ranging in size from 200-260 beds, were collected from nine medical-surgical units. A case was defined as an adult patient who fell on one of the 24 study units during the study period as reported in the hospital incident reporting system. In this study, a fall was defined as an unintentional change in position which resulted in coming to rest on the ground or a lower level. Falls resulting from catastrophic clinical events (e.g., seizure, stroke, or arrhythmia) were excluded. For each case, we identified up to two controls with a similar hospital length of stay that were on the same nursing unit at the same time the case fell. The data collection was approved by the Institutional Review Board of the University of Tennessee Health Science Center. This secondary analysis was approved by the University of Florida Institutional Review Board.

For peer review only

Exposures

Exposures were determined for both cases and controls using medical records review by personnel blinded to the case status of the patient. We designated an index time for the controls based on the date and time that the matched case fell. This index time was used to establish the timeframe for collecting the exposures. Specifically, we recorded exposures most proximate to the time of the fall, or the index time for controls. Additionally, we excluded exposures that were greater than 24 hours before the fall event. Our primary exposures were serum sodium and other abnormal laboratory values that may indicate volume depletion including high BUN, creatinine, BUN to creatinine ratio, and hematocrit. Volume depletion occurs when there is a decrease in circulating blood volume which can occur as a result of inadequate fluid intake or excessive loss of fluids or blood. The abnormal laboratory value classifications can be found in Table 2[36].

Covariates

Demographic covariates in this study included age, race, and gender. We also adjusted for the medical conditions of Parkinson's disease, dementia, hypertension, congestive heart failure (CHF), diabetes mellitus, and stroke. Presence of these comorbid conditions was defined as a positive history in the medical record. Also, we controlled for whether the patient had experienced an acute mental status change within the past twenty-four hours prior to the fall index time. Additionally, we controlled for the patient's fall risk score prior to the fall index time. This score is based on the Morse Fall Scale with the inclusion of medications which potentially effect mobility or cognition (e.g., sedatives, antipsychotics, antidepressants, diuretics, and opiates)[11]. We based the binary measure of high fall risk (Yes/No) on this standardized tool which is used across the four hospitals.

Statistical Analysis

For peer review only

Data analysis was performed using SAS software, Version 9.4 of the SAS System for Windows (SAS Institute, Inc., Cary, NC). Univariate descriptive statistics were calculated by fall status for the overall sample. Descriptive statistics including mean and standard deviation were calculated for continuous data, and counts and percentages were calculated for categorical data. Potential multicollinearity issues were evaluated by testing the correlation between categorical exposures using the phi coefficient. Further, multicollinearity diagnostics were evaluated by checking condition index and variable inflation values. Since cases either had one or two controls, and were matched based on potential confounders, conditional logistic regression with m:n matching was used to determine bivariate and multivariable associations[39, 40]. Using this approach, the adjusted odds ratios were estimated along with their corresponding 95% confidence intervals. A series of multivariable models were created. Each multivariable model included one of the abnormal laboratory values of interest and the potential covariates. Potential covariates were included in the multivariable models regardless of bivariate *P*-values.

Additional analyses were performed on serum sodium levels to further analyze the potential relationship between hyponatremia and hospital-acquired falls. A categorical variable was created which included three levels of serum sodium including 125mEq/L or lower, 126mEq/L to 134mEq/L, and 135mEq/L or greater. These categories were created to determine if there was a dose-response association between hospital-acquired falls and sodium levels. Conditional logistic regression with m:n matching was used to determine the bivariate and multivariable associations. Using this approach, the unadjusted and adjusted odds ratios were estimated along with their corresponding 95% confidence intervals. Two models were created including (1) an unadjusted model which only included the categorical sodium level variable and (2) an adjusted model which included the categorical sodium level variable and the covariates of

For peer review only

high BUN, high creatinine, high BUN to creatinine ratio, age, race, gender, high fall risk score, acute mental status change, and the medical conditions of dementia, hypertension, CHF, diabetes, and stroke.

For peer review only

RESULTS

The final sample included 699 fallers and 1189 matched controls. Descriptive statistics by fall status for the final sample can be found in Table 1. Controls were 62 years old on average and 59.7% female whereas the cases were 65 years old on average and 50.9% female. Additionally, only 35.8% of controls compared to 55.7% of cases were assessed as high risk prior to the fall index time. Also, 75.4% of cases had a diagnosis of hypertension whereas 68.5% of controls had a diagnosis of hypertension. We did not include the exposures of Parkinson's Disease, high hematocrit, and high sodium in further analyses due to low frequencies of less than 3% in this sample.

Table 1. Characteristics of cases and controls by Fall Status (N=1888)

	Controls (n=1189)			Cases (n=699)			
**Factor**	**N**	**Frequency (%)**	**Mean (SD)**	**N**	**Frequency (%)**	**Mean (SD)**	***P*** [a]
Age	1189		61.6 (17.8)	699		64.9 (16.1)	<0.01
Race	1189			698			<0.01
White		538 (45.3)			392 (56.2)		
Not White		651 (54.8)			306 (43.8)		
Gender	1189			698			<0.01
Male		479 (40.3)			343 (49.1)		
Female		710 (59.7)			355 (50.9)		
Hospital	1187			699			0.979[b]
University		528 (44.5)			320 (45.8)		
Community 1		285 (24.0)			164 (23.5)		
Community 2		97 (8.2)			57 (8.2)		
Community 3		277 (23.3)			158 (22.6)		
High fall risk	1169	419 (35.8)		684	381 (55.7)		<0.01
Mental status change	1183	224 (18.9)		692	180 (26.0)		<0.01
Medical conditions							
Parkinson's	1185	12 (1.0)		698	12 (1.7)		0.14
Dementia	1185	139 (11.7)		698	106 (15.2)		0.06
Hypertension	1188	814 (68.5)		699	527 (75.4)		<0.01
CHF	1186	252 (21.3)		694	160 (23.1)		0.35
Diabetes	1186	394 (33.2)		696	261 (37.5)		0.07
Stroke	1185	143 (12.1)		695	108 (15.5)		0.03

Note. CHF = Congestive heart failure.
[a]*P* values were determined using conditional logistic regression with m:n matching
[b]Cases and controls were matched by unit and therefore matched by hospital as well

For peer review only

Unadjusted odds ratios from bivariate conditional logistic regression models can be found in Table 2. In the unadjusted model, the presence of low sodium increased the odds of a hospital-acquired fall (odds ratio (OR)=1.485, 95% confidence interval (CI)=1.136-1.940). In contrast, the presence of elevated BUN levels decreased the odds of a hospital-acquired fall (OR=0.785, 95% CI=0.622-0.991). The presence of elevated creatinine levels and BUN to creatinine ratios were not significantly associated with the occurrence of a hospital-acquired fall in the unadjusted models.

Table 2. Bivariate and Multivariable Conditional Logistic Regression of Abnormal Blood Urea Nitrogen (BUN), Creatinine, and Sodium Values on Hospital-Acquired Falls

Model	Factor	Controls (n)	Cases (n)	Unadjusted OR (95% CI)	Adjusted OR[a] (95% CI)
1	BUN > 21mg/dL	337	213	0.785 (0.622, 0.991)	0.643 (0.494, 0.838)
	BUN ≤ 21mg/dL	579	411	Reference	Reference
2	Cr > 1.1mg/dL[b]	363	230	0.827 (0.655, 1.045)	0.700 (0.535, 0.916)
	Cr ≤ 1.1mg/dL[c]	556	397	Reference	Reference
3	BUN/Cr > 20	215	140	0.860 (0.657, 1.124)	0.766 (0.578, 1.042)
	BUN/Cr ≤ 20	700	483	Reference	Reference
4	Na ≥ 135mEq/L	762	469	Reference	Reference
	Na < 135mEq/L	155	148	1.485 (1.136, 1.940)	1.356 (1.013, 1.816)

Note. BUN = Blood Urea Nitrogen, Cr = Creatinine, Na = Sodium.
[a]Conditional logistic regression model adjusted for age, race, gender, high fall risk score, acute mental status change, and medical conditions of dementia, hypertension, CHF, diabetes, and stroke
[b]In males > 1.2mg/dL
[c]In males ≤ 1.2mg/dL

Adjusted odds ratios from the four multivariable conditional logistic regression models can also be found in Table 2. Each of these models included one abnormal laboratory value which may indicate volume depletion while controlling for the covariates of age, race, gender, high fall risk score, acute mental status change, and the medical conditions of dementia, hypertension, CHF, diabetes, and stroke. After adjusting for these covariates, the presence of low sodium significantly increased the odds of a hospital-acquired fall by 36% (*P*=0.04). Presence of

For peer review only

high BUN and high creatinine were significantly associated with decreased odds of experiencing a hospital-acquired fall (OR = 0.643, *P*=0.001, and OR=0.700, *P*=0.009, respectively). However, a high BUN to creatinine ratio was not significantly associated with experiencing a hospital-acquired fall in the adjusted model. The results of these four models indicate that based on abnormal laboratory values, volume depletion may not be associated with increased odds of a hospital-acquired fall.

Categories of serum sodium levels including 125mEq/L or lower, 126mEq/L to 134mEq/L, and 135mEq/L or greater were analyzed further. Adjusted and unadjusted odds ratios from conditional logistic regression models for these categories of serum sodium levels can be found in Table 3. Two models were created including an unadjusted model and a model adjusted for presence of high BUN, high creatinine, a high BUN to creatinine ratio, age, race, gender, high fall risk score, acute mental status change, and the medical conditions of dementia, hypertension, CHF, diabetes, and stroke. In the unadjusted model, those with a serum sodium level of 125mEq/L or lower were 4.82 times more likely to experience a hospital-acquired fall as compared to a serum sodium level of 135mEq/L or greater (95% CI= 1.54-15.12). In the adjusted model, those with a serum sodium level of 125mEq/L or lower were 5.08 (95% CI=1.43-18.08) times more likely to experience a hospital-acquired fall.

Table 3. Bivariate and Multivariable Conditional Logistic Regression of Serum Sodium Levels on Hospital-Acquired Falls

Serum Sodium (mEq/L)	Controls (n)	Cases (n)	Unadjusted OR (95% CI)	OR Adjusted for multiple risk factors[a] (95% CI)
125 or lower	5	12	4.82 (1.54-15.12)	5.08 (1.43-18.08)
126-134	150	136	1.37 (1.04-1.81)	1.27 (0.94-1.73)
135 or greater	762	469	Reference	Reference

[a]Conditional logistic regression model adjusted for high blood urea nitrogen (BUN), high creatinine, high BUN to creatinine ratio, age, race, gender, high fall risk score, acute mental status change, and medical conditions of dementia, hypertension, CHF, diabetes, and stroke

For peer review only

Additionally, Figure 1 displays the proportion of cases (fallers) and controls (non-fallers) among the three categories of serum sodium levels. Of those with a serum sodium level of 125mEq/L or lower, approximately 71% were fallers and 29% were non-fallers. In contrast, of those with a serum sodium level of 126mEq/L to 134mEq/L and 135mEq/L or greater, approximately 48% and 38% were fallers and 52% and 62% were non-fallers, respectively.

For peer review only

DISCUSSION

In this study, we identified important associations between patient-level risk factors and hospital-acquired falls. Of the four variables chosen as indicators of volume depletion that were usable in the analyses, only low sodium was independently related to increased odds for falling in the hospital setting. However, the abnormal laboratory values of high BUN levels and high creatinine levels were significantly associated with decreased odds of experiencing a hospital-acquired fall. It is possible that high BUN and creatinine levels appear to be protective because high BUN and creatinine levels can result in fatigue[36]. A fatigued patient might ambulate less and therefore be less likely to experience a fall. Also, it should be noted that high BUN and creatinine levels can be indicative of conditions other than volume depletion, such as a hypercatabolic state. In addition to potentially indicating volume depletion, electrolyte abnormalities such as low sodium can occur secondary to other conditions such as syndrome of inappropriate antidiuretic hormone secretion (SIADH). Hyponatremia can result in deficits including altered cognitive status and weakness[10, 36]. Lacking significant positive associations between other volume depletion-related laboratory values, it is likely that the relationship between hyponatremia and hospital-acquired falls is related to deficits arising from hyponatremia rather than directly related to volume depletion. Additionally, we observed that the odds of experiencing a hospital-acquired fall appear to increase as sodium levels decrease independent of high BUN, high creatinine, a high BUN to creatinine ratio, and other risk factors. This pattern further suggests that hyponatremia is a risk factor for experiencing a hospital-acquired fall and that volume depletion does not appear to be the casual pathway.

Our findings add to previous work examining the relationship between hyponatremia and hospital-acquired falls. An observational study conducted in a single hospital in Japan with a

For peer review only

sample containing 97 fallers found that when controlling for age, comorbidities, and increases in sedative doses, hyponatremia (serum sodium < 135 mEq/L) significantly increased the odds of experiencing a hospital-acquired fall (OR = 1.751)[25]. Additionally, an observational study examining hyponatremia and hospital-acquired falls among a psychiatric population found that when controlling for age, antiepileptic drug use, and selective serotonin re-uptake inhibitor (SSRI) use, hyponatremia significantly increased the odds of experiencing a hospital-acquired fall (OR = 4.38)[24]. In contrast, a case-control study found that low sodium was not significantly associated with experiencing a hospital-acquired fall in a population of only those age 65 and older[23]. However, this study was limited by sample size with only 62 fallers and 62 controls which may have limited the statistical power of its findings. Collectively, it is not easy to determine the relative contribution of our work because not all of these studies provided an operational definition of hyponatremia.

The relationship between hyponatremia and non-hospital-acquired falls has also been examined in an observational study of fall-related and non-fall related geriatric trauma admissions. The investigators of that study determined that when controlling for potential confounders such as age and pre-existing conditions, patients with fall-related admissions were significantly more likely to have low sodium levels (OR=1.81)[41]. Also, investigators using a case-control study design including patients admitted to the emergency room with and without chronic hyponatremia, found that patients with chronic hyponatremia were significantly more likely to have experienced a fall after controlling for covariates (OR=67)[42]. In a similar case-control study including geriatric patients that were admitted with and without hyponatremia, investigators found that when controlling for covariates such as age, gender, admitting diagnosis, and medications, patients with hyponatremia were significantly more likely to have a fall

For peer review only

associated with their admission (e.g., as a presenting complaint) (OR=3.12)[43]. Additionally, investigators examining hyponatremia among community-dwelling older adults found that after adjusting for age, gender, and diuretic use, persons with hyponatremia were significantly more likely to have experienced a fall (P=0.01)[44].

Unlike most previous work related to laboratory values and falls, our study is informed by a conceptual framework that hypothesized the relationship between laboratory values and fall risk[38]. Additional strengths of this study include the matching strategy and large sample size. Matching was based on nursing unit, date and time of fall, and length of stay which should control for environmental and staffing factors at the time of the fall. In previous research, measures of the hospital environment such as physical measures (e.g., unit layout), resource availability measures (e.g., nurse staffing), and culture measures (e.g., magnet or teaching status) have been significantly associated with patient outcomes[45-47]. Additionally, at the unit environment level, researchers have found that some medical units tend to have persistently low fall rates whereas other medical units have persistently high fall rates[47]. The environment appears to have an effect on patient falls and it is therefore important to account for this variation. However, it should also be noted that our design cannot control for all environmental factors such as location of patient room relative to the nursing station.

A limitation of this study is that only laboratory values were used as potential indicators of volume depletion. Further, the abnormal laboratory values of low sodium, high BUN, high creatinine, and a high BUN to creatinine ratio are not specific to volume depletion, but can also potentially indicate conditions other than volume depletion. For instance, hyponatremia can occur as a result of syndrome of inappropriate antidiuretic hormone secretion (SIADH). Additionally, and elevated BUN, creatinine, and BUN to creatinine ratio levels can be seen in

For peer review only

conditions such as congestive heart failure, sepsis, gastrointestinal obstruction, and internal bleeding[35]. Although these abnormal laboratory values are not specific to volume depletion, they do have clinical value in determining whether a patient is volume depleted. For instance, increases in blood urea levels have been significantly associated with decreased hydration status[34]. Additionally, if volume depletion is caused vomiting, diarrhea, diuretics, or adrenal insufficiency, then the patient is likely to also be hyponatremic[29]. No laboratory value gold standard exists for identifying volume depletion, however the laboratory values used in this study are commonly used in clinical practice and have been used in prior research as markers for volume depletion[30-33, 35]. Future work to further examine the potential relationship between volume depletion and hospital-acquired falls should consider using other potential indicators such as urine output, urine specific gravity or orthostatic blood pressure. However, laboratory values are frequently collected in the hospital setting and are relatively reliable measures that have been neglected in previous falls research.

Additional limitations include that this was a secondary data analysis. This limited us to the exposure data available in the existing dataset. Also, this study is limited by selecting for only those hospitalized patients who received laboratory results within their last 24 hours of hospitalization prior to the fall index time. This, as well as sampling from one area of the country, limits the generalizability of these findings. However, the sample for this study was collected from four hospitals which contributes to the generalizability of these findings. Additional limitations include that observational research is susceptible to threats to internal validity including the incomplete control of potential confounders. However, we believe that our matching strategy helped to control for the relevant potential confounders of length of stay, nurse staffing, unit culture, and unit environment.

For peer review only

CONCLUSION

In this matched case-control study, we found a strong relationship between hyponatremia and fall risk in hospitalized patients. This relationship is independent of increased BUN, creatinine, and BUN to creatinine ratio as well as independent of demographic risk factors and other patient-level risk factors for hospital falls. Conversely, we found no other associations of laboratory findings consistent with volume depletion and increased risk of falls. It is possible that other indicators of volume depletion, such as orthostatic hypotension, are necessary for the examination of this relationship. The results of this study do indicate that abnormal laboratory values, such as low sodium, can be useful for identifying hospitalized patients at increased risk of experiencing a fall. Symptoms associated with hyponatremia, including mental status changes, can be addressed with system- and patient-level interventions such as modifying the patient environment and regular patient surveillance. Further investigation into abnormal laboratory values as predictors of hospitalized-acquired falls is warranted, and if validated, should be added to currently utilized fall risk scales.

For peer review only

Funding:
This work was supported by the National Institute on Aging (R01-AG033005 to R.I.S.); and the National Center for Advancing Translational Sciences of the National Institutes of Health under University of Florida Clinical and Translational Science Awards (TL1TR001428 and UL1TR001427 to E.A.F.).

The content is solely the responsibility of the authors and does not necessarily represent the official views of the National Institutes of Health.

Competing Interests:
All authors have completed the ICMJE uniform disclosure form at www.icmje.org/coi_disclosure.pdf and declare: no support from any organization for the submitted work other than government funding which is specified in the Funding statement; no financial relationships with any organizations that might have an interest in the submitted work in the previous three years; no other relationships or activities that could appear to have influenced the submitted work except that RIS serves as an expert witness in cases of hospital falls.

Author Contributions:
All authors were involved in formulating the study concept/design. AMC, PAR, and RIS were involved in the acquisition of the data and EAF, MTW, and RIS performed the statistical analysis. EAF, RJL, MTW, AMM, LCM, and RIS were involved in the interpretation of the data, and all of the authors participated in the preparation of this manuscript. Additionally, all authors approved of the submitted manuscript.

For peer review only

REFERENCES

1. Centers for Disease Control and Prevention. Falls among older adults: An overview. https://www.cdc.gov/HomeandRecreationalSafety/Falls/adultfalls.html (Accessed June 2015).

2. Ganz DA, Huang C, Saliba D, et al. Preventing falls in hospitals: a toolkit for improving quality of care. (Prepared by RAND Corporation, Boston University School of Public Health, and ECRI Institute under Contract No. HHSA290201000017I TO #1.). Rockville, MD: Agency for Healthcare Research and Quality 2013. https://www.ahrq.gov/sites/default/files/publications/files/fallpxtoolkit.pdf (Accessed June 2015).

3. Angalakuditi M, Coley KC, Kirisci L, et al. A case-control study to assess the impact of anemia and other risk factors for in-hospital falls. *J Patient Saf* 2007;3(1):16-21. doi: 10.1097/pts.0b013e318030c2ff

4. Hendrich A, Nyhuis A, Kippenbrock T, et al. Hospital falls: development of a predictive model for clinical practice. *Appl Nurs Res* 1995;8(3):129-39.

5. Hendrich AL, Bender PS, Nyhuis A. Validation of the Hendrich II Fall Risk Model: a large concurrent case/control study of hospitalized patients. *Appl Nurs Res* 2003;16(1):9-21. doi: 10.1053/apnr.2003.YAPNR2

6. Krauss MJ, Evanoff B, Hitcho E, et al. A case-control study of patient, medication, and care-related risk factors for inpatient falls. *J Gen Intern Med* 2005;20(2):116-22. doi: 10.1111/j.1525-1497.2005.40171.x

7. Yauk S, Hopkins BA, Phillips CD, et al. Predicting in-hospital falls: development of the Scott and White Falls Risk Screener. *J Nurs Care Qual* 2005;20(2):128-33.

For peer review only

8. Morse JM, Tylko SJ, Dixon HA. Characteristics of the fall-prone patient. *Gerontologist* 1987;27(4):516-22.

9. Ash KL, MacLeod P, Clark L. A case control study of falls in the hospital setting. *J Gerontol Nurs* 1998;24(12):7-15.

10. Rao SS. Prevention of falls in older patients. *Am Fam Physician* 2005;72(1):81-8.

11. Morse JM, Morse RM, Tylko SJ. Development of a scale to identify the fall-prone patient. *Can J Aging* 1989;8(4):366-77. doi: 10.1017/s0714980800008576

12. Oliver D, Britton M, Seed P, et al. Development and evaluation of evidence based risk assessment tool (STRATIFY) to predict which elderly inpatients will fall: case-control and cohort studies. *BMJ* 1997;315(7115):1049-53.

13. Aranda-Gallardo M, Morales-Asencio JM, Canca-Sanchez JC, et al. Instruments for assessing the risk of falls in acute hospitalized patients: a systematic review and meta-analysis. *BMC Health Serv Res* 2013;13(1):122. doi: 10.1186/1472-6963-13-122

14. Chen YC, Chien SF, Chen LK. Risk factors associated with falls among Chinese hospital inpatients in Taiwan. *Arch Gerontol Geriatr* 2009;48(2):132-6. doi: 10.1016/j.archger.2007.11.006

15. Plati C, Lanara V, Mantas J. Risk factors responsible for patients' falls. *Scand J Caring Sci* 1992;6(2):113-8.

16. Dharmarajan TS, Avula S, Norkus EP. Anemia increases risk for falls in hospitalized older adults: an evaluation of falls in 362 hospitalized, ambulatory, long-term care, and community patients. *J Am Med Dir Assoc* 2006;7(5):287-93. doi: 10.1016/j.jamda.2005.10.010

For peer review only

17. O'Hagan C, O'Connell B. The relationship between patient blood pathology values and patient falls in an acute-care setting: a retrospective analysis. *Int J Nurs Pract* 2005;11(4):161-8. doi: 10.1111/j.1440-172X.2005.00519.x

18. Church S, Robinson TN, Angles EM, et al. Postoperative falls in the acute hospital setting: characteristics, risk factors, and outcomes in males. *Am J Surg* 2011;201(2):197-202. doi: 10.1016/j.amjsurg.2009.12.013

19. Capone LJ, Albert NM, Bena JF, et al. Predictors of a fall event in hospitalized patients with cancer. *Oncol Nurs Forum* 2012;39(5):E407-15. doi: 10.1188/12.onf.e407-e415

20. Byers V, Arrington ME, Finstuen K. Predictive risk factors associated with stroke patient falls in acute care settings. *J Neurosci Nurs* 1990;22(3):147-54.

21. Stenvall M, Olofsson B, Lundstrom M, et al. Inpatient falls and injuries in older patients treated for femoral neck fracture. *Arch Gerontol Geriatr* 2006;43(3):389-99. doi: 10.1016/j.archger.2006.01.004

22. Chu LW, Pei CK, Chiu A, et al. Risk factors for falls in hospitalized older medical patients. *J Gerontol A Biol Sci Med Sci* 1999;54(1):M38-43.

23. Walker PC, Alrawi A, Mitchell JF, et al. Medication use as a risk factor for falls among hospitalized elderly patients. *Am J Health Syst Pharm* 2005;62(23):2495-9. doi: 10.2146/ajhp050116

24. Bun S, Serby MJ, Friedmann P. Psychotropic medication use, hyponatremia, and falls in an inpatient population: a retrospective study. *J Clin Psychopharmacol* 2011;31(3):395-7.

25. Tachi T, Yokoi T, Goto C, et al. Hyponatremia and hypokalemia as risk factors for falls. *Eur J Clin Nutr* 2015;69(2):205-10. doi: 10.1038/ejcn.2014.195

For peer review only

26. Robinson SB, Rosher RB. Can a beverage cart help improve hydration? *Geriatr Nurs* 2002;23(4):208-11. doi: 10.1067/mgn.2002.126967

27. Rubenstein LZ. Falls in older people: epidemiology, risk factors and strategies for prevention. *Age and Ageing* 2006;35:37-41. doi: 10.1093/ageing/afl084

28. Ungar A, Rafanelli M, Iacomelli I, et al. Fall prevention in the elderly. *Clin Cases Miner Bone Metab* 2013;10(2):91-5.

29. Morley JE. Dehydration, Hypernatremia, and Hyponatremia. 2015;31(3):389-99. doi: 10.1016/j.cger.2015.04.007

30. Gross CR, Lindquist RD, Woolley AC, et al. Clinical indicators of dehydration severity in elderly patients. *J Emerg Med* 1992;10(3):267-74.

31. McGee S, Abernethy WB, Simel DL. Is this patient hypovolemic? *JAMA* 1999;281(11):1022-9.

32. Sinert R, Spektor M. Clinical assessment of hypovolemia. *Ann Emerg Med* 2005;45(3):327-9. doi: 10.1016/j.annemergmed.2004.09.021

33. Riccardi A, Chiarbonello B, Minuto P, et al. Identification of the hydration state in emergency patients: correlation between caval index and BUN/creatinine ratio. *Eur Rev Med Pharmacol Sci* 2013;17(13):1800-3.

34. Shepherd J, Hatfield S, Kilpatrick ES. Is there still a role for measuring serum urea in an age of eGFR? Evidence of its use when assessing patient hydration. *Nephron Clin Pract* 2009;113(3):c203-6. doi: 10.1159/000233057

35. Robinson BE, Weber H. Dehydration despite drinking: beyond the BUN/Creatinine ratio. *J Am Med Dir Assoc* 2004;5(2 Suppl):S67-71. doi: 10.1097/01.JAM.0000035860.71185.39

For peer review only

36. Van Leeuwen AM, Bladh ML. Davis's Comprehensive Handbook of Laboratory & Diagnostic Tests with Nursing Implications. 6th ed. Philadelphia, PA: F.A. Davis Co. 2015.

37. Simon EE. Hyponatremia Evaluation and Treatment. New York, NY: Springer 2013.

38. Choi YS, Lawler E, Boenecke CA, et al. Developing a multi-systemic fall prevention model, incorporating the physical environment, the care process and technology: a systematic review. *J Adv Nurs* 2011;67(12):2501-24. doi: 10.1111/j.1365-2648.2011.05672.x

39. SAS Institute Inc. SAS/STAT 9.2 User's Guide. 2nd ed., Conditional logisitc regression for matched pairs data. Cary, NC: SAS Institute Inc. 2009.

40. Allison PD. Logistic Regression Using SAS: Theory and Application. 2nd ed. Cary, NC: SAS Institute Inc. 2012. https://support.sas.com/documentation/cdl/en/statug/63033/HTML/default/viewer.htm#statug_logistic_sect062.htm (Accessed August 2016).

41. Rittenhouse KJ, To T, Rogers A, et al. Hyponatremia as a fall predictor in a geriatric trauma population. *Injury* 2015;46(1):119-23. doi: 10.1016/j.injury.2014.06.013

42. Renneboog B, Musch W, Vandemergel X, et al. Mild chronic hyponatremia is associated with falls, unsteadiness, and attention deficits. *Am J Med* 2006;119(1):71.e1-8. doi: 10.1016/j.amjmed.2005.09.026

43. Ahamed S, Anpalahan M, Savvas S, et al. Hyponatraemia in older medical patients: implications for falls and adverse outcomes of hospitalisation. *Intern Med J* 2014;44(10):991-97. doi: 10.1111/imj.12535

For peer review only

44. Gunathilake R, Oldmeadow C, McEvoy M, et al. Mild hyponatremia is associated with impaired cognition and falls in community-dwelling older persons. *J Am Geriatr Soc* 2013;61(10):1838-9. doi: 10.1111/jgs.12468

45. Lake ET, Shang J, Klaus S, et al. Patient falls: Association with hospital Magnet status and nursing unit staffing. *Res Nurs Health* 2010;33(5):413-25. doi: 10.1002/nur.20399

46. Everhart D, Schumacher JR, Duncan RP, et al. Determinants of hospital fall rate trajectory groups: a longitudinal assessment of nurse staffing and organizational characteristics. *Health Care Manage Rev* 2014;39(4):352-60. doi: 10.1097/HMR.0000000000000013

47. Staggs VS, Mion LC, Shorr RI. Consistent differences in medical unit fall rates: implications for research and practice. *J Am Geriatr Soc* 2015;63(5):983-7. doi: 10.1111/jgs.13387

For peer review only

CAPTIONS

Figure 1. Proportions of Cases and Controls Among Serum Sodium Levels.

This figure displays three categories of serum sodium levels including 125mEq/L or lower, 126mEq/L to 134mEq/L, and 135mEq/L or greater. Of those cases and controls with these three different serum sodium levels, the black bars indicate the proportion that are cases and the grey bars indicate the proportion that are controls. Included at the bottom of the figure, is the number of cases and controls within each category of serum sodium level.

For peer review only

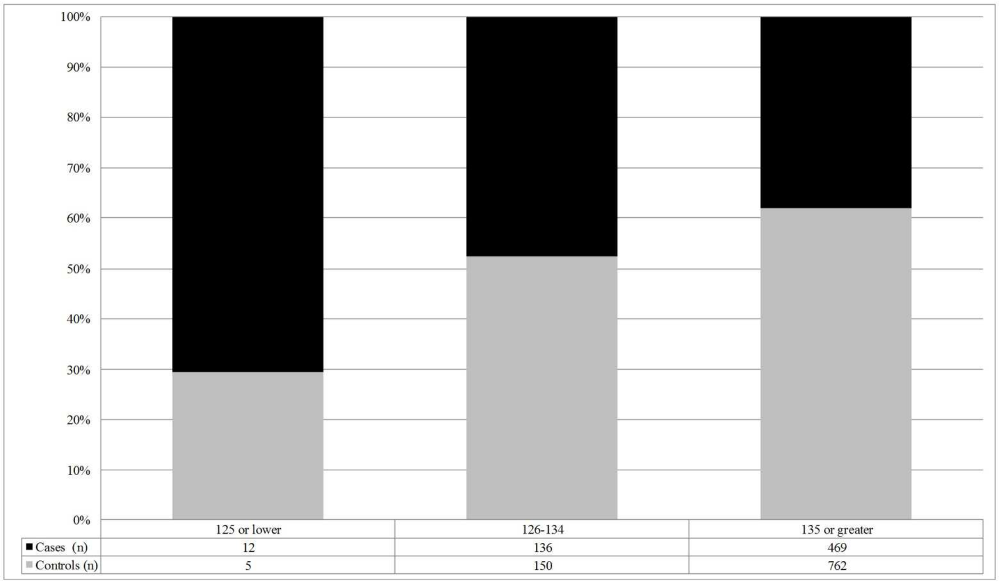

Figure 1. Proportions of Cases and Controls Among Serum Sodium Levels.
This figure displays three categories of serum sodium levels including 125mEq/L or lower, 126mEq/L to 134mEq/L, and 135mEq/L or greater. Of those cases and controls with these three different serum sodium levels, the black bars indicate the proportion that are cases and the grey bars indicate the proportion that are controls. Included at the bottom of the figure, is the number of cases and controls within each category of serum sodium level.

239x139mm (300 x 300 DPI)

STROBE 2007 (v4) Statement—Checklist of items that should be included in reports of *case-control studies*

Section/Topic	Item #	Recommendation	Reported on page #
**Title and abstract**	1	(*a*) Indicate the study's design with a commonly used term in the title or the abstract	1-2
		(*b*) Provide in the abstract an informative and balanced summary of what was done and what was found	2
**Introduction**			
Background/rationale	2	Explain the scientific background and rationale for the investigation being reported	5-6
Objectives	3	State specific objectives, including any prespecified hypotheses	6
**Methods**			
Study design	4	Present key elements of study design early in the paper	7-8
Setting	5	Describe the setting, locations, and relevant dates, including periods of recruitment, exposure, follow-up, and data collection	7
Participants	6	(*a*) Give the eligibility criteria, and the sources and methods of case ascertainment and control selection. Give the rationale for the choice of cases and controls	7
		(*b*) For matched studies, give matching criteria and the number of controls per case	7
Variables	7	Clearly define all outcomes, exposures, predictors, potential confounders, and effect modifiers. Give diagnostic criteria, if applicable	7-8, 11
Data sources/ measurement	8*	For each variable of interest, give sources of data and details of methods of assessment (measurement). Describe comparability of assessment methods if there is more than one group	7-8, 11
Bias	9	Describe any efforts to address potential sources of bias	7
Study size	10	Explain how the study size was arrived at	n/a, based on number of falls occurring during study period-pg7
Quantitative variables	11	Explain how quantitative variables were handled in the analyses. If applicable, describe which groupings were chosen and why	8-9
Statistical methods	12	(*a*) Describe all statistical methods, including those used to control for confounding	8-9
		(*b*) Describe any methods used to examine subgroups and interactions	n/a
		(*c*) Explain how missing data were addressed	n/a, large sample size

For peer review only

		(*d*) If applicable, explain how matching of cases and controls was addressed	8
		(*e*) Describe any sensitivity analyses	n/a
**Results**			
Participants	13*	(a) Report numbers of individuals at each stage of study—eg numbers potentially eligible, examined for eligibility, confirmed eligible, included in the study, completing follow-up, and analysed	10
		(b) Give reasons for non-participation at each stage	n/a, secondary analysis
		(c) Consider use of a flow diagram	n/a
Descriptive data	14*	(a) Give characteristics of study participants (eg demographic, clinical, social) and information on exposures and potential confounders	10-11
		(b) Indicate number of participants with missing data for each variable of interest	10-11
Outcome data	15*	Report numbers in each exposure category, or summary measures of exposure	10-12
Main results	16	(*a*) Give unadjusted estimates and, if applicable, confounder-adjusted estimates and their precision (eg, 95% confidence interval). Make clear which confounders were adjusted for and why they were included	11-12
		(*b*) Report category boundaries when continuous variables were categorized	11-12
		(*c*) If relevant, consider translating estimates of relative risk into absolute risk for a meaningful time period	n/a
Other analyses	17	Report other analyses done—eg analyses of subgroups and interactions, and sensitivity analyses	n/a
**Discussion**			
Key results	18	Summarise key results with reference to study objectives	14
Limitations	19	Discuss limitations of the study, taking into account sources of potential bias or imprecision. Discuss both direction and magnitude of any potential bias	16-17
Interpretation	20	Give a cautious overall interpretation of results considering objectives, limitations, multiplicity of analyses, results from similar studies, and other relevant evidence	14-17
Generalisability	21	Discuss the generalisability (external validity) of the study results	16-17
**Other information**			
Funding	22	Give the source of funding and the role of the funders for the present study and, if applicable, for the original study on which the present article is based	18

For peer review only

BMJ Open

Associations Between Hyponatremia, Volume Depletion, and the Risk of Falls in US Hospitalized Patients: A Case-Control Study

Journal:	*BMJ Open*
Manuscript ID	bmjopen-2017-017045.R2
Article Type:	Research
Date Submitted by the Author:	29-May-2017
Complete List of Authors:	Fehlberg, Elizabeth; University of Florida, College of Nursing; University of Florida, Clinical and Translational Science Institute<br>Lucero, Robert; University of Florida, College of Nursing; Malcom Randall VA Medical Center, Center for Innovation on Disability and Rehabilitation Research<br>Weaver, Michael; University of Florida, College of Nursing<br>McDaniel, Anna; University of Florida, College of Nursing<br>Chandler, A.; Methodist Healthcare University Hospital<br>Richey, Phyllis; University of Tennessee Health Science Center, Department of Preventative Medicine<br>Mion, Lorraine; The Ohio State University, College of Nursing<br>Shorr, Ronald; Malcom Randall Veterans Affairs Medical Center, Geriatric Research Education and Clinical Center; University of Florida, Epidemiology
**Primary Subject Heading**:	Epidemiology
Secondary Subject Heading:	Nursing, Geriatric medicine
Keywords:	EPIDEMIOLOGY, GERIATRIC MEDICINE, Clinical chemistry < PATHOLOGY, Hospital related, Risk factors, Falls

SCHOLARONE™
Manuscripts

For peer review only

Associations Between Hyponatremia, Volume Depletion, and the Risk of Falls in US Hospitalized Patients: A Case-Control Study

Elizabeth A. Fehlberg, BSN, RN [1,2]
Robert J. Lucero, PhD, MPH, RN, FAAN [1,2,3]
Michael T. Weaver PhD, RN, FAAN [1]
Anna M. McDaniel PhD, RN, FAAN [1]
A. Michelle Chandler [4]
Phyllis A. Richey, PhD [5]
Lorraine C. Mion PhD, RN, FAAN [6]
Ronald I. Shorr MD, MS, FACP [2,7,8]

1. University of Florida College of Nursing; Gainesville, FL
2. University of Florida Clinical and Translational Science Institute; Gainesville, FL
3. Center for Innovation on Disability and Rehabilitation Research (CINDRR), Malcom Randall VAMC; Gainesville, FL
4. Methodist Healthcare University Hospital; Memphis, TN
5. Department of Preventive Medicine, University of Tennessee Health Science Center; Memphis, TN
6. College of Nursing, The Ohio State University; Columbus, OH
7. Geriatric Research Education and Clinical Centers (GRECC), Malcom Randall VAMC; Gainesville, FL
8. Department of Epidemiology, University of Florida; Gainesville, FL

Corresponding Author:
Elizabeth A. Fehlberg
Health Professions, Nursing and Pharmacy Complex
1225 Center Drive
PO Box 100197
Gainesville, FL 32610-0197
352-273-6370; Fax: 352-273-6568
efehlberg@ufl.edu

Word Count: 3307

For peer review only

ABSTRACT

OBJECTIVES: We aimed to determine if abnormal laboratory values which may indicate volume depletion are associated with increased odds of experiencing a hospital-acquired fall.

DESIGN: Matched case-control.

SETTING: Four hospitals located in the southeast United States.

PARTICIPANTS: Data from 699 adult fallers and 1189 matched controls (non-fallers) were collected via chart review from 2005 to 2010. Controls were matched to cases by nursing unit, time of fall, and length of stay.

OUTCOME MEASURES: The primary exposures included serum sodium, blood urea nitrogen (BUN), creatinine, BUN/creatinine ratio, and hematocrit. Conditional logistic regression with m:n matching was used to determine adjusted and unadjusted odds ratios.

RESULTS: Serum sodium levels were strongly associated with falls. In models controlling for demographic and other fall risk factors, patients with serum sodium levels of 125mEq/L or less were associated with increased odds of experiencing a fall as compared to those with serum sodium levels of greater than 134mEq/L (adjusted odds ratio (aOR) = 5.08, 95% confidence interval (CI)=1.43-18.08). Conversely, elevated BUN, creatinine, and elevated BUN/creatinine ratios were not associated with increased odds of experiencing a fall (aOR=0.64, 95% CI=0.49-0.84, and aOR=0.70, 95% CI=0.54-0.92, and aOR=0.77, 95% CI=0.58-1.04, respectively.)

CONCLUSIONS: Laboratory indices that may indicate volume depletion appear to be unrelated to falls. However, hyponatremia does appear to be a risk factor for falls, and those with serum sodium levels below 126mEq/L are at especially high risk. It may be that other deficits associated with hyponatremia, such as altered mental status, are associated with risk of experiencing a hospital-acquired fall. These results indicate that abnormal laboratory values,

For peer review only

such as low sodium, can be useful for identifying hospitalized patients at risk of falling. Therefore, further investigation into abnormal laboratory values as predictors of hospital-acquired falls is warranted.

Key words: *epidemiology, geriatric medicine, clinical chemistry, hospital related, risk factors, falls*

For peer review only

Strengths and limitations of this study

- The main strengths of this study include: 1) a large sample size collected over six years, 2) the setting which included four hospitals, and 3) a matching strategy that controls for environmental and staffing factors at the time of a fall event.
- Only laboratory values that are not specific for volume depletion were used as potential indicators of volume depletion instead of other indicators such as urine output or orthostatic blood pressure.
- Our design did not account for all environmental factors such as location of patient room relative to the nursing station.
- Due to the nature of observational research, we cannot exclude the possibility of incomplete control of potential confounders and there were small numbers of patients with serum sodium levels of 125mEq/L or less.
- The generalizability of our findings is limited by the context of our study and the sampling frame which relied on only patients whose laboratory results were known 24 hours prior to the fall index time.

For peer review only

INTRODUCTION

Each year between 700,000 and 1,000,000 falls occur in United States hospitals with associated total direct medical costs of $34 billion[1, 2]. Additionally, falls are the leading cause of both fatal and nonfatal injuries among older adults[1]. Numerous studies have been conducted to examine the associations between patient-level risk factors and hospital-acquired falls. Examples of previously identified risk factors include dementia, diabetes, depression, cognitive status, and impaired mobility[3-9]. Although many researchers have examined demographic factors and health conditions, few studies have aimed to understand the underlying biological mechanisms related to falling. Laboratory values, which have generally been neglected in previous falls research, can potentially increase our understanding of risk factors for hospital-acquired falls[10].

Researchers have created tools to assist in the identification of patients at risk for falls including the Morse Fall Scale, the STRATIFY Scale, and the Hendrich Fall Risk Model[4, 11, 12]. These tools include risk factors such as history of falls, mobility impairments, and altered elimination. However, the predictability of these tools has been shown to vary considerably when subjected to external validations[13]. This indicates that there is potential for improvement which could be achieved with the inclusion of additional factors such as laboratory values. Researchers have examined the diagnosis of anemia, hemoglobin levels, and/or hematocrit levels as potential risk factors for hospital-acquired falls[3, 8, 14-22]. However, there has been limited examination of other laboratory values. Previous research has examined hyponatremia in a psychiatric population, hyponatremia and hypokalemia in a Japanese cohort, albumin in a postoperative population, and creatinine, sodium, and the ratio of blood urea nitrogen (BUN) to creatinine[18, 23-25]. Additionally, one study examined bivariate associations between

For peer review only

approximately thirty laboratory values and falling[17]. However, all of the aforementioned non-anemia laboratory work was limited in sample size, with approximately 100 fallers.

Previous research has identified volume depletion as a risk factor for falling, but to the best of our knowledge there has been limited examination of volume depletion and hospital-acquired falls. We found one study that examined the relationship between fluid intake and falls in a nursing home and found a significant decline in falls (P=0.05) during a hydration program[26]. However, further examination of this potential relationship is warranted, especially since nursing home and hospital populations differ. Abnormal serum creatinine, BUN, sodium, and hematocrit levels can indicate multiple health conditions, including volume depletion[10, 27-35]. Specifically, abnormal laboratory values which may indicate volume depletion include high BUN levels (>21mg/dL), high creatinine levels (>1.1mg/dL in females, >1.2mg/dL in males), high BUN to creatinine ratios (>20), high hematocrit levels (>45% in females and >51% in males), or high and low sodium levels (>145mEq/L and <135mEq/L, respectively)[36, 37]. To address whether volume depletion is potentially related to hospital-acquired falls, we used a case-control design to test if abnormal BUN, creatinine, hematocrit, and sodium levels were associated with odds of a hospital-acquired fall.

For peer review only

METHODS

Conceptual Framework

This study is informed by Choi's conceptual multi-systemic fall prevention model[38]. Intrinsic risk factors, including physiological factors (i.e., laboratory values), are directly associated with fall risk. In addition, symptoms secondary to physiological changes are associated with environmental and technological interventions. By discovering physiological intrinsic risk factors, modifications to the environment and care processes can theoretically lead to a reduction in falls.

Study Population

This case-control study was conducted using data that were collected between 2005 and 2010 across four hospitals. These hospitals are located in the southeastern United States and belong to the same health system. Data from the largest hospital, a 635-bed urban community facility, were collected from fifteen medical-surgical units. Data from the three other suburban community hospitals, ranging in size from 200-260 beds, were collected from nine medical-surgical units. A case was defined as an adult patient who fell on one of the 24 study units during the study period as reported in the hospital incident reporting system. In this study, a fall was defined as an unintentional change in position which resulted in coming to rest on the ground or a lower level. Falls resulting from catastrophic clinical events (e.g., seizure, stroke, or arrhythmia) were excluded. For each case, we identified up to two controls with a similar hospital length of stay that were on the same nursing unit at the same time the case fell. The data collection was approved by the Institutional Review Board of the University of Tennessee Health Science Center. This secondary analysis was approved by the University of Florida Institutional Review Board.

For peer review only

Exposures

Exposures were determined for both cases and controls using medical records review by personnel blinded to the case status of the patient. We designated an index time for the controls based on the date and time that the matched case fell. This index time was used to establish the timeframe for collecting the exposures. Specifically, we recorded exposures most proximate to the time of the fall, or the index time for controls. Additionally, we excluded exposures that were greater than 24 hours before the fall event. Our primary exposures were serum sodium and other abnormal laboratory values that may indicate volume depletion including high BUN, creatinine, BUN to creatinine ratio, and hematocrit. Volume depletion occurs when there is a decrease in circulating blood volume which can occur as a result of inadequate fluid intake or excessive loss of fluids or blood. The abnormal laboratory value classifications can be found in Table 2[36].

Covariates

Demographic covariates in this study included age, race, and gender. We also adjusted for the medical conditions of Parkinson's disease, dementia, hypertension, congestive heart failure (CHF), diabetes mellitus, and stroke. Presence of these comorbid conditions was defined as a positive history in the medical record. Also, we controlled for whether the patient had experienced an acute mental status change within the past twenty-four hours prior to the fall index time. Additionally, we controlled for the patient's fall risk score prior to the fall index time. This score is based on the Morse Fall Scale with the inclusion of medications which potentially effect mobility or cognition (e.g., sedatives, antipsychotics, antidepressants, diuretics, and opiates)[11]. We based the binary measure of high fall risk (Yes/No) on this standardized tool which is used across the four hospitals.

Statistical Analysis

For peer review only

Data analysis was performed using SAS software, Version 9.4 of the SAS System for Windows (SAS Institute, Inc., Cary, NC). Univariate descriptive statistics were calculated by fall status for the overall sample. Descriptive statistics including mean and standard deviation were calculated for continuous data, and counts and percentages were calculated for categorical data. Potential multicollinearity issues were evaluated by testing the correlation between categorical exposures using the phi coefficient. Further, multicollinearity diagnostics were evaluated by checking condition index and variable inflation values. Since cases either had one or two controls, and were matched based on potential confounders, conditional logistic regression with m:n matching was used to determine bivariate and multivariable associations[39, 40]. Using this approach, the adjusted odds ratios were estimated along with their corresponding 95% confidence intervals. A series of multivariable models were created. Each multivariable model included one of the abnormal laboratory values of interest and the potential covariates. Potential covariates were included in the multivariable models regardless of bivariate *P*-values.

Additional analyses were performed on serum sodium levels to further analyze the potential relationship between hyponatremia and hospital-acquired falls. A categorical variable was created which included three levels of serum sodium including 125mEq/L or lower, 126mEq/L to 134mEq/L, and 135mEq/L or greater. These categories were created to determine if there was a dose-response association between hospital-acquired falls and sodium levels. Conditional logistic regression with m:n matching was used to determine the bivariate and multivariable associations. Using this approach, the unadjusted and adjusted odds ratios were estimated along with their corresponding 95% confidence intervals. Two models were created including (1) an unadjusted model which only included the categorical sodium level variable and (2) an adjusted model which included the categorical sodium level variable and the covariates of

For peer review only

high BUN, high creatinine, high BUN to creatinine ratio, age, race, gender, high fall risk score, acute mental status change, and the medical conditions of dementia, hypertension, CHF, diabetes, and stroke.

For peer review only

RESULTS

The final sample included 699 fallers and 1189 matched controls. Descriptive statistics by fall status for the final sample can be found in Table 1. Controls were 62 years old on average and 59.7% female whereas the cases were 65 years old on average and 50.9% female. Additionally, only 35.8% of controls compared to 55.7% of cases were assessed as high risk prior to the fall index time. Also, 75.4% of cases had a diagnosis of hypertension whereas 68.5% of controls had a diagnosis of hypertension. We did not include the exposures of Parkinson's Disease, high hematocrit, and high sodium in further analyses due to low frequencies of less than 3% in this sample. Specifically, 1.72% of cases and 1.01% of controls had a diagnosis of

Table 1. Characteristics of cases and controls by Fall Status (N=1888)

	Controls (n=1189)			Cases (n=699)			
Factor	N	Frequency (%)	Mean (SD)	N	Frequency (%)	Mean (SD)	*P* [a]
Age	1189		61.6 (17.8)	699		64.9 (16.1)	<0.01
Race	1189			698			<0.01
White		538 (45.3)			392 (56.2)		
Not White		651 (54.8)			306 (43.8)		
Gender	1189			698			<0.01
Male		479 (40.3)			343 (49.1)		
Female		710 (59.7)			355 (50.9)		
Hospital	1187			699			0.979[b]
University		528 (44.5)			320 (45.8)		
Community 1		285 (24.0)			164 (23.5)		
Community 2		97 (8.2)			57 (8.2)		
Community 3		277 (23.3)			158 (22.6)		
High fall risk	1169	419 (35.8)		684	381 (55.7)		<0.01
Mental status change	1183	224 (18.9)		692	180 (26.0)		<0.01
Medical conditions							
Parkinson's	1185	12 (1.0)		698	12 (1.7)		0.14
Dementia	1185	139 (11.7)		698	106 (15.2)		0.06
Hypertension	1188	814 (68.5)		699	527 (75.4)		<0.01
CHF	1186	252 (21.3)		694	160 (23.1)		0.35
Diabetes	1186	394 (33.2)		696	261 (37.5)		0.07
Stroke	1185	143 (12.1)		695	108 (15.5)		0.03

Note. CHF = Congestive heart failure.
[a]*P* values were determined using conditional logistic regression with m:n matching
[b]Cases and controls were matched by unit and therefore matched by hospital as well

For peer review only

Parkinson's Disease, 1.45% of cases and 1.09% of controls had high hematocrit levels, and 2.11% of cases and 2.40% of controls had high sodium levels.

Unadjusted odds ratios from bivariate conditional logistic regression models can be found in Table 2. In the unadjusted model, the presence of low sodium increased the odds of a hospital-acquired fall (odds ratio (OR)=1.485, 95% confidence interval (CI)=1.136-1.940). In contrast, the presence of elevated BUN levels decreased the odds of a hospital-acquired fall (OR=0.785, 95% CI=0.622-0.991). The presence of elevated creatinine levels and BUN to creatinine ratios were not significantly associated with the occurrence of a hospital-acquired fall in the unadjusted models.

Table 2. Bivariate and Multivariable Conditional Logistic Regression of Abnormal Blood Urea Nitrogen (BUN), Creatinine, and Sodium Values on Hospital-Acquired Falls

**Model**	**Factor**	**Controls (n)**	**Cases (n)**	**Unadjusted OR (95% CI)**	**Adjusted OR[a] (95% CI)**
1	BUN > 21mg/dL	337	213	0.785 (0.622, 0.991)	0.643 (0.494, 0.838)
	BUN ≤ 21mg/dL	579	411	Reference	Reference
2	Cr > 1.1mg/dL[b]	363	230	0.827 (0.655, 1.045)	0.700 (0.535, 0.916)
	Cr ≤ 1.1mg/dL[c]	556	397	Reference	Reference
3	BUN/Cr > 20	215	140	0.860 (0.657, 1.124)	0.766 (0.578, 1.042)
	BUN/Cr ≤ 20	700	483	Reference	Reference
4	Na ≥ 135mEq/L	762	469	Reference	Reference
	Na < 135mEq/L	155	148	1.485 (1.136, 1.940)	1.356 (1.013, 1.816)

Note. BUN = Blood Urea Nitrogen, Cr = Creatinine, Na = Sodium.
[a]Conditional logistic regression model adjusted for age, race, gender, high fall risk score, acute mental status change, and medical conditions of dementia, hypertension, CHF, diabetes, and stroke
[b]In males > 1.2mg/dL
[c]In males ≤ 1.2mg/dL

Adjusted odds ratios from the four multivariable conditional logistic regression models can also be found in Table 2. Each of these models included one abnormal laboratory value which may indicate volume depletion while controlling for the covariates of age, race, gender, high fall risk score, acute mental status change, and the medical conditions of dementia,

For peer review only

hypertension, CHF, diabetes, and stroke. After adjusting for these covariates, the presence of low sodium significantly increased the odds of a hospital-acquired fall by 36% (*P*=0.04). Presence of high BUN and high creatinine were significantly associated with decreased odds of experiencing a hospital-acquired fall (OR = 0.643, *P*=0.001, and OR=0.700, *P*=0.009, respectively). However, a high BUN to creatinine ratio was not significantly associated with experiencing a hospital-acquired fall in the adjusted model. The results of these four models indicate that based on abnormal laboratory values, volume depletion may not be associated with increased odds of a hospital-acquired fall.

Categories of serum sodium levels including 125mEq/L or lower, 126mEq/L to 134mEq/L, and 135mEq/L or greater were analyzed further. Adjusted and unadjusted odds ratios from conditional logistic regression models for these categories of serum sodium levels can be found in Table 3. Two models were created including an unadjusted model and a model adjusted for presence of high BUN, high creatinine, a high BUN to creatinine ratio, age, race, gender, high fall risk score, acute mental status change, and the medical conditions of dementia, hypertension, CHF, diabetes, and stroke. In the unadjusted model, those with a serum sodium level of 125mEq/L or lower were 4.82 times more likely to experience a hospital-acquired fall as compared to a serum sodium level of 135mEq/L or greater (95% CI= 1.54-15.12). In the adjusted

Table 3. Bivariate and Multivariable Conditional Logistic Regression of Serum Sodium Levels on Hospital-Acquired Falls

Serum Sodium (mEq/L)	Controls (n)	Cases (n)	Unadjusted OR (95% CI)	OR Adjusted for multiple risk factors[a] (95% CI)
125 or lower	5	12	4.82 (1.54-15.12)	5.08 (1.43-18.08)
126-134	150	136	1.37 (1.04-1.81)	1.27 (0.94-1.73)
135 or greater	762	469	Reference	Reference

[a]Conditional logistic regression model adjusted for high blood urea nitrogen (BUN), high creatinine, high BUN to creatinine ratio, age, race, gender, high fall risk score, acute mental status change, and medical conditions of dementia, hypertension, CHF, diabetes, and stroke

For peer review only

model, those with a serum sodium level of 125mEq/L or lower were 5.08 (95% CI=1.43-18.08) times more likely to experience a hospital-acquired fall. Additionally, Figure 1 displays the proportion of cases (fallers) and controls (non-fallers) among the three categories of serum sodium levels. Of those with a serum sodium level of 125mEq/L or lower, approximately 71% were fallers and 29% were non-fallers. In contrast, of those with a serum sodium level of 126mEq/L to 134mEq/L and 135mEq/L or greater, approximately 48% and 38% were fallers and 52% and 62% were non-fallers, respectively.

For peer review only

DISCUSSION

In this study, we identified important associations between patient-level risk factors and hospital-acquired falls. Of the four variables chosen as indicators of volume depletion that were usable in the analyses, only low sodium was independently related to increased odds for falling in the hospital setting. However, the abnormal laboratory values of high BUN levels and high creatinine levels were significantly associated with decreased odds of experiencing a hospital-acquired fall. It is possible that high BUN and creatinine levels appear to be protective because high BUN and creatinine levels can result in fatigue[36]. A fatigued patient might ambulate less and therefore be less likely to experience a fall. Also, it should be noted that high BUN and creatinine levels can be indicative of conditions other than volume depletion, such as a hypercatabolic state. In addition to potentially indicating volume depletion, electrolyte abnormalities such as low sodium can occur secondary to other conditions such as syndrome of inappropriate antidiuretic hormone secretion (SIADH). Hyponatremia can result in deficits including altered cognitive status and weakness[10, 36]. Lacking significant positive associations between other volume depletion-related laboratory values, it is likely that the relationship between hyponatremia and hospital-acquired falls is related to deficits arising from hyponatremia rather than directly related to volume depletion. Additionally, we observed that the odds of experiencing a hospital-acquired fall appear to increase as sodium levels decrease independent of high BUN, high creatinine, a high BUN to creatinine ratio, and other risk factors. This pattern further suggests that hyponatremia is a risk factor for experiencing a hospital-acquired fall and that volume depletion does not appear to be the casual pathway.

Our findings add to previous work examining the relationship between hyponatremia and hospital-acquired falls. An observational study conducted in a single hospital in Japan with a

For peer review only

sample containing 97 fallers found that when controlling for age, comorbidities, and increases in sedative doses, hyponatremia (serum sodium < 135 mEq/L) significantly increased the odds of experiencing a hospital-acquired fall (OR = 1.751)[25]. Additionally, an observational study examining hyponatremia and hospital-acquired falls among a psychiatric population found that when controlling for age, antiepileptic drug use, and selective serotonin re-uptake inhibitor (SSRI) use, hyponatremia significantly increased the odds of experiencing a hospital-acquired fall (OR = 4.38)[24]. In contrast, a case-control study found that low sodium was not significantly associated with experiencing a hospital-acquired fall in a population of only those age 65 and older[23]. However, this study was limited by sample size with only 62 fallers and 62 controls which may have limited the statistical power of its findings. Collectively, it is not easy to determine the relative contribution of our work because not all of these studies provided an operational definition of hyponatremia.

The relationship between hyponatremia and non-hospital-acquired falls has also been examined in an observational study of fall-related and non-fall related geriatric trauma admissions. The investigators of that study determined that when controlling for potential confounders such as age and pre-existing conditions, patients with fall-related admissions were significantly more likely to have low sodium levels (OR=1.81)[41]. Also, investigators using a case-control study design including patients admitted to the emergency room with and without chronic hyponatremia, found that patients with chronic hyponatremia were significantly more likely to have experienced a fall after controlling for covariates (OR=67)[42]. In a similar case-control study including geriatric patients that were admitted with and without hyponatremia, investigators found that when controlling for covariates such as age, gender, admitting diagnosis, and medications, patients with hyponatremia were significantly more likely to have a fall

For peer review only

associated with their admission (e.g., as a presenting complaint) (OR=3.12)[43]. Additionally, investigators examining hyponatremia among community-dwelling older adults found that after adjusting for age, gender, and diuretic use, persons with hyponatremia were significantly more likely to have experienced a fall (P=0.01)[44].

Unlike most previous work related to laboratory values and falls, our study is informed by a conceptual framework that hypothesized the relationship between laboratory values and fall risk[38]. Additional strengths of this study include the matching strategy and large sample size. Matching was based on nursing unit, date and time of fall, and length of stay which should control for environmental and staffing factors at the time of the fall. In previous research, measures of the hospital environment such as physical measures (e.g., unit layout), resource availability measures (e.g., nurse staffing), and culture measures (e.g., magnet or teaching status) have been significantly associated with patient outcomes[45-47]. Additionally, at the unit environment level, researchers have found that some medical units tend to have persistently low fall rates whereas other medical units have persistently high fall rates[47]. The environment appears to have an effect on patient falls and it is therefore important to account for this variation. However, it should also be noted that our design cannot control for all environmental factors such as location of patient room relative to the nursing station.

A limitation of this study is that only laboratory values were used as potential indicators of volume depletion. Further, the abnormal laboratory values of low sodium, high BUN, high creatinine, and a high BUN to creatinine ratio are not specific to volume depletion, but can also potentially indicate conditions other than volume depletion. For instance, hyponatremia can occur as a result of syndrome of inappropriate antidiuretic hormone secretion (SIADH). Additionally, elevated BUN, creatinine, and BUN to creatinine ratio levels can be seen in

For peer review only

conditions such as congestive heart failure, sepsis, gastrointestinal obstruction, and internal bleeding[35]. Although these abnormal laboratory values are not specific to volume depletion, they do have clinical value in determining whether a patient is volume depleted. For instance, increases in blood urea levels have been significantly associated with decreased hydration status[34]. Additionally, if volume depletion is caused by vomiting, diarrhea, diuretics, or adrenal insufficiency, then the patient is likely to also be hyponatremic[29]. No laboratory value gold standard exists for identifying volume depletion, however the laboratory values used in this study are commonly used in clinical practice and have been used in prior research as markers for volume depletion[30-33, 35]. Future work to further examine the potential relationship between volume depletion and hospital-acquired falls should consider using other potential indicators such as urine output, urine specific gravity or orthostatic blood pressure. However, laboratory values are frequently collected in the hospital setting and are relatively reliable measures that have been neglected in previous falls research.

Additional limitations include that this was a secondary data analysis. This limited us to the exposure data available in the existing dataset. Also, this study is limited by selecting for only those hospitalized patients who received laboratory results within their last 24 hours of hospitalization prior to the fall index time. This, as well as sampling from one area of the country, limits the generalizability of these findings. However, the sample for this study was collected from four hospitals which contributes to the generalizability of these findings. Additional limitations include that observational research is susceptible to threats to internal validity including the incomplete control of potential confounders. However, we believe that our matching strategy helped to control for the relevant potential confounders of length of stay, nurse staffing, unit culture, and unit environment.

For peer review only

CONCLUSION

In this matched case-control study, we found a strong relationship between hyponatremia and fall risk in hospitalized patients. This relationship is independent of increased BUN, creatinine, and BUN to creatinine ratio as well as independent of demographic risk factors and other patient-level risk factors for hospital falls. Conversely, we found no other associations of laboratory findings consistent with volume depletion and increased risk of falls. It is possible that other indicators of volume depletion, such as orthostatic hypotension, are necessary for the examination of this relationship. The results of this study do indicate that abnormal laboratory values, such as low sodium, can be useful for identifying hospitalized patients at increased risk of experiencing a fall. Symptoms associated with hyponatremia, including mental status changes, can be addressed with system- and patient-level interventions such as modifying the patient environment and regular patient surveillance. Further investigation into abnormal laboratory values as predictors of hospitalized-acquired falls is warranted, and if validated, should be added to currently utilized fall risk scales.

For peer review only

Funding:

This work was supported by the National Institute on Aging (R01-AG033005 to R.I.S.); and the National Center for Advancing Translational Sciences of the National Institutes of Health under University of Florida Clinical and Translational Science Awards (TL1TR001428 and UL1TR001427 to E.A.F.).

The content is solely the responsibility of the authors and does not necessarily represent the official views of the National Institutes of Health.

Competing Interests:

All authors have completed the ICMJE uniform disclosure form at www.icmje.org/coi_disclosure.pdf and declare: no support from any organization for the submitted work other than government funding which is specified in the Funding statement; no financial relationships with any organizations that might have an interest in the submitted work in the previous three years; no other relationships or activities that could appear to have influenced the submitted work except that RIS serves as an expert witness in cases of hospital falls.

Author Contributions:

All authors were involved in formulating the study concept/design. AMC, PAR, and RIS were involved in the acquisition of the data and EAF, MTW, and RIS performed the statistical analysis. EAF, RJL, MTW, AMM, LCM, and RIS were involved in the interpretation of the data, and all of the authors participated in the preparation of this manuscript. Additionally, all authors approved of the submitted manuscript.

For peer review only

REFERENCES

1. Centers for Disease Control and Prevention. Falls among older adults: An overview. https://www.cdc.gov/HomeandRecreationalSafety/Falls/adultfalls.html (Accessed June 2015).

2. Ganz DA, Huang C, Saliba D, et al. Preventing falls in hospitals: a toolkit for improving quality of care. (Prepared by RAND Corporation, Boston University School of Public Health, and ECRI Institute under Contract No. HHSA290201000017I TO #1.). Rockville, MD: Agency for Healthcare Research and Quality 2013. https://www.ahrq.gov/sites/default/files/publications/files/fallpxtoolkit.pdf (Accessed June 2015).

3. Angalakuditi M, Coley KC, Kirisci L, et al. A case-control study to assess the impact of anemia and other risk factors for in-hospital falls. *J Patient Saf* 2007;3(1):16-21. doi: 10.1097/pts.0b013e318030c2ff

4. Hendrich A, Nyhuis A, Kippenbrock T, et al. Hospital falls: development of a predictive model for clinical practice. *Appl Nurs Res* 1995;8(3):129-39.

5. Hendrich AL, Bender PS, Nyhuis A. Validation of the Hendrich II Fall Risk Model: a large concurrent case/control study of hospitalized patients. *Appl Nurs Res* 2003;16(1):9-21. doi: 10.1053/apnr.2003.YAPNR2

6. Krauss MJ, Evanoff B, Hitcho E, et al. A case-control study of patient, medication, and care-related risk factors for inpatient falls. *J Gen Intern Med* 2005;20(2):116-22. doi: 10.1111/j.1525-1497.2005.40171.x

7. Yauk S, Hopkins BA, Phillips CD, et al. Predicting in-hospital falls: development of the Scott and White Falls Risk Screener. *J Nurs Care Qual* 2005;20(2):128-33.

For peer review only

8. Morse JM, Tylko SJ, Dixon HA. Characteristics of the fall-prone patient. *Gerontologist* 1987;27(4):516-22.

9. Ash KL, MacLeod P, Clark L. A case control study of falls in the hospital setting. *J Gerontol Nurs* 1998;24(12):7-15.

10. Rao SS. Prevention of falls in older patients. *Am Fam Physician* 2005;72(1):81-8.

11. Morse JM, Morse RM, Tylko SJ. Development of a scale to identify the fall-prone patient. *Can J Aging* 1989;8(4):366-77. doi: 10.1017/s0714980800008576

12. Oliver D, Britton M, Seed P, et al. Development and evaluation of evidence based risk assessment tool (STRATIFY) to predict which elderly inpatients will fall: case-control and cohort studies. *BMJ* 1997;315(7115):1049-53.

13. Aranda-Gallardo M, Morales-Asencio JM, Canca-Sanchez JC, et al. Instruments for assessing the risk of falls in acute hospitalized patients: a systematic review and meta-analysis. *BMC Health Serv Res* 2013;13(1):122. doi: 10.1186/1472-6963-13-122

14. Chen YC, Chien SF, Chen LK. Risk factors associated with falls among Chinese hospital inpatients in Taiwan. *Arch Gerontol Geriatr* 2009;48(2):132-6. doi: 10.1016/j.archger.2007.11.006

15. Plati C, Lanara V, Mantas J. Risk factors responsible for patients' falls. *Scand J Caring Sci* 1992;6(2):113-8.

16. Dharmarajan TS, Avula S, Norkus EP. Anemia increases risk for falls in hospitalized older adults: an evaluation of falls in 362 hospitalized, ambulatory, long-term care, and community patients. *J Am Med Dir Assoc* 2006;7(5):287-93. doi: 10.1016/j.jamda.2005.10.010

For peer review only

17. O'Hagan C, O'Connell B. The relationship between patient blood pathology values and patient falls in an acute-care setting: a retrospective analysis. *Int J Nurs Pract* 2005;11(4):161-8. doi: 10.1111/j.1440-172X.2005.00519.x

18. Church S, Robinson TN, Angles EM, et al. Postoperative falls in the acute hospital setting: characteristics, risk factors, and outcomes in males. *Am J Surg* 2011;201(2):197-202. doi: 10.1016/j.amjsurg.2009.12.013

19. Capone LJ, Albert NM, Bena JF, et al. Predictors of a fall event in hospitalized patients with cancer. *Oncol Nurs Forum* 2012;39(5):E407-15. doi: 10.1188/12.onf.e407-e415

20. Byers V, Arrington ME, Finstuen K. Predictive risk factors associated with stroke patient falls in acute care settings. *J Neurosci Nurs* 1990;22(3):147-54.

21. Stenvall M, Olofsson B, Lundstrom M, et al. Inpatient falls and injuries in older patients treated for femoral neck fracture. *Arch Gerontol Geriatr* 2006;43(3):389-99. doi: 10.1016/j.archger.2006.01.004

22. Chu LW, Pei CK, Chiu A, et al. Risk factors for falls in hospitalized older medical patients. *J Gerontol A Biol Sci Med Sci* 1999;54(1):M38-43.

23. Walker PC, Alrawi A, Mitchell JF, et al. Medication use as a risk factor for falls among hospitalized elderly patients. *Am J Health Syst Pharm* 2005;62(23):2495-9. doi: 10.2146/ajhp050116

24. Bun S, Serby MJ, Friedmann P. Psychotropic medication use, hyponatremia, and falls in an inpatient population: a retrospective study. *J Clin Psychopharmacol* 2011;31(3):395-7.

25. Tachi T, Yokoi T, Goto C, et al. Hyponatremia and hypokalemia as risk factors for falls. *Eur J Clin Nutr* 2015;69(2):205-10. doi: 10.1038/ejcn.2014.195

For peer review only

26. Robinson SB, Rosher RB. Can a beverage cart help improve hydration? *Geriatr Nurs* 2002;23(4):208-11. doi: 10.1067/mgn.2002.126967

27. Rubenstein LZ. Falls in older people: epidemiology, risk factors and strategies for prevention. *Age and Ageing* 2006;35:37-41. doi: 10.1093/ageing/afl084

28. Ungar A, Rafanelli M, Iacomelli I, et al. Fall prevention in the elderly. *Clin Cases Miner Bone Metab* 2013;10(2):91-5.

29. Morley JE. Dehydration, Hypernatremia, and Hyponatremia. 2015;31(3):389-99. doi: 10.1016/j.cger.2015.04.007

30. Gross CR, Lindquist RD, Woolley AC, et al. Clinical indicators of dehydration severity in elderly patients. *J Emerg Med* 1992;10(3):267-74.

31. McGee S, Abernethy WB, Simel DL. Is this patient hypovolemic? *JAMA* 1999;281(11):1022-9.

32. Sinert R, Spektor M. Clinical assessment of hypovolemia. *Ann Emerg Med* 2005;45(3):327-9. doi: 10.1016/j.annemergmed.2004.09.021

33. Riccardi A, Chiarbonello B, Minuto P, et al. Identification of the hydration state in emergency patients: correlation between caval index and BUN/creatinine ratio. *Eur Rev Med Pharmacol Sci* 2013;17(13):1800-3.

34. Shepherd J, Hatfield S, Kilpatrick ES. Is there still a role for measuring serum urea in an age of eGFR? Evidence of its use when assessing patient hydration. *Nephron Clin Pract* 2009;113(3):c203-6. doi: 10.1159/000233057

35. Robinson BE, Weber H. Dehydration despite drinking: beyond the BUN/Creatinine ratio. *J Am Med Dir Assoc* 2004;5(2 Suppl):S67-71. doi: 10.1097/01.JAM.0000035860.71185.39

For peer review only

36. Van Leeuwen AM, Bladh ML. Davis's Comprehensive Handbook of Laboratory & Diagnostic Tests with Nursing Implications. 6th ed. Philadelphia, PA: F.A. Davis Co. 2015.

37. Simon EE. Hyponatremia Evaluation and Treatment. New York, NY: Springer 2013.

38. Choi YS, Lawler E, Boenecke CA, et al. Developing a multi-systemic fall prevention model, incorporating the physical environment, the care process and technology: a systematic review. *J Adv Nurs* 2011;67(12):2501-24. doi: 10.1111/j.1365-2648.2011.05672.x

39. SAS Institute Inc. SAS/STAT 9.2 User's Guide. 2nd ed., Conditional logisitc regression for matched pairs data. Cary, NC: SAS Institute Inc. 2009.

40. Allison PD. Logistic Regression Using SAS: Theory and Application. 2nd ed. Cary, NC: SAS Institute Inc. 2012. https://support.sas.com/documentation/cdl/en/statug/63033/HTML/default/viewer.htm#statug_logistic_sect062.htm (Accessed August 2016).

41. Rittenhouse KJ, To T, Rogers A, et al. Hyponatremia as a fall predictor in a geriatric trauma population. *Injury* 2015;46(1):119-23. doi: 10.1016/j.injury.2014.06.013

42. Renneboog B, Musch W, Vandemergel X, et al. Mild chronic hyponatremia is associated with falls, unsteadiness, and attention deficits. *Am J Med* 2006;119(1):71.e1-8. doi: 10.1016/j.amjmed.2005.09.026

43. Ahamed S, Anpalahan M, Savvas S, et al. Hyponatraemia in older medical patients: implications for falls and adverse outcomes of hospitalisation. *Intern Med J* 2014;44(10):991-97. doi: 10.1111/imj.12535

For peer review only

44. Gunathilake R, Oldmeadow C, McEvoy M, et al. Mild hyponatremia is associated with impaired cognition and falls in community-dwelling older persons. *J Am Geriatr Soc* 2013;61(10):1838-9. doi: 10.1111/jgs.12468

45. Lake ET, Shang J, Klaus S, et al. Patient falls: Association with hospital Magnet status and nursing unit staffing. *Res Nurs Health* 2010;33(5):413-25. doi: 10.1002/nur.20399

46. Everhart D, Schumacher JR, Duncan RP, et al. Determinants of hospital fall rate trajectory groups: a longitudinal assessment of nurse staffing and organizational characteristics. *Health Care Manage Rev* 2014;39(4):352-60. doi: 10.1097/HMR.0000000000000013

47. Staggs VS, Mion LC, Shorr RI. Consistent differences in medical unit fall rates: implications for research and practice. *J Am Geriatr Soc* 2015;63(5):983-7. doi: 10.1111/jgs.13387

For peer review only

CAPTIONS

Figure 1. Proportions of Cases and Controls Among Serum Sodium Levels.

This figure displays three categories of serum sodium levels including 125mEq/L or lower, 126mEq/L to 134mEq/L, and 135mEq/L or greater. Of those cases and controls with these three different serum sodium levels, the black bars indicate the proportion that are cases and the grey bars indicate the proportion that are controls. Included at the bottom of the figure, is the number of cases and controls within each category of serum sodium level.

For peer review only

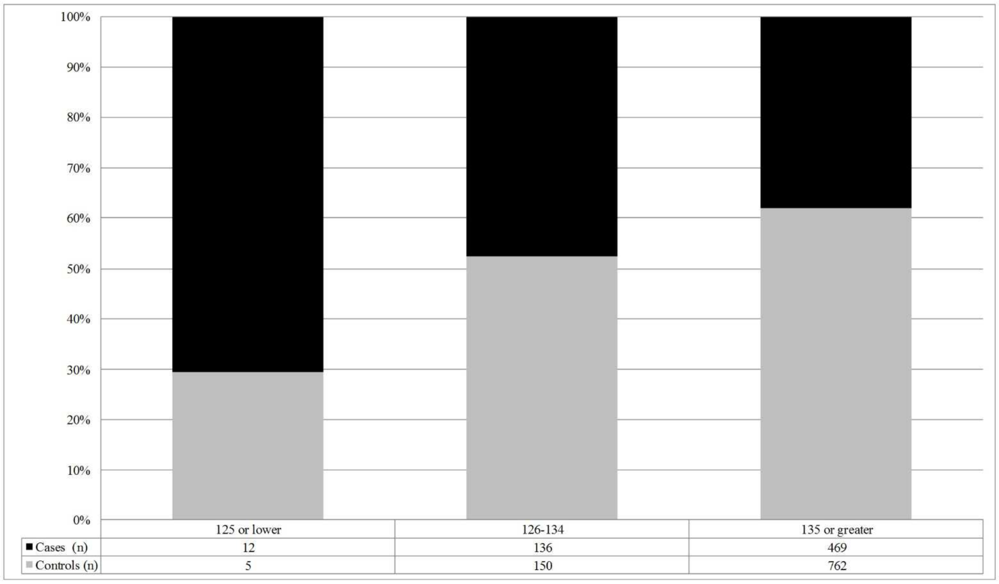

Figure 1. Proportions of Cases and Controls Among Serum Sodium Levels.
This figure displays three categories of serum sodium levels including 125mEq/L or lower, 126mEq/L to 134mEq/L, and 135mEq/L or greater. Of those cases and controls with these three different serum sodium levels, the black bars indicate the proportion that are cases and the grey bars indicate the proportion that are controls. Included at the bottom of the figure, is the number of cases and controls within each category of serum sodium level.

239x139mm (300 x 300 DPI)

iew only

STROBE 2007 (v4) Statement—Checklist of items that should be included in reports of *case-control studies*

Section/Topic	Item #	Recommendation	Reported on page #
**Title and abstract**	1	(*a*) Indicate the study's design with a commonly used term in the title or the abstract	1-2
		(*b*) Provide in the abstract an informative and balanced summary of what was done and what was found	2
**Introduction**			
Background/rationale	2	Explain the scientific background and rationale for the investigation being reported	5-6
Objectives	3	State specific objectives, including any prespecified hypotheses	6
**Methods**			
Study design	4	Present key elements of study design early in the paper	7-8
Setting	5	Describe the setting, locations, and relevant dates, including periods of recruitment, exposure, follow-up, and data collection	7
Participants	6	(*a*) Give the eligibility criteria, and the sources and methods of case ascertainment and control selection. Give the rationale for the choice of cases and controls	7
		(*b*) For matched studies, give matching criteria and the number of controls per case	7
Variables	7	Clearly define all outcomes, exposures, predictors, potential confounders, and effect modifiers. Give diagnostic criteria, if applicable	7-8, 11
Data sources/ measurement	8*	For each variable of interest, give sources of data and details of methods of assessment (measurement). Describe comparability of assessment methods if there is more than one group	7-8, 11
Bias	9	Describe any efforts to address potential sources of bias	7
Study size	10	Explain how the study size was arrived at	n/a, based on number of falls occurring during study period-pg7
Quantitative variables	11	Explain how quantitative variables were handled in the analyses. If applicable, describe which groupings were chosen and why	8-9
Statistical methods	12	(*a*) Describe all statistical methods, including those used to control for confounding	8-9
		(*b*) Describe any methods used to examine subgroups and interactions	n/a
		(*c*) Explain how missing data were addressed	n/a, large sample size

For peer review only

		(*d*) If applicable, explain how matching of cases and controls was addressed	8
		(*e*) Describe any sensitivity analyses	n/a
**Results**			
Participants	13*	(a) Report numbers of individuals at each stage of study—eg numbers potentially eligible, examined for eligibility, confirmed eligible, included in the study, completing follow-up, and analysed	10
		(b) Give reasons for non-participation at each stage	n/a, secondary analysis
		(c) Consider use of a flow diagram	n/a
Descriptive data	14*	(a) Give characteristics of study participants (eg demographic, clinical, social) and information on exposures and potential confounders	10-11
		(b) Indicate number of participants with missing data for each variable of interest	10-11
Outcome data	15*	Report numbers in each exposure category, or summary measures of exposure	10-12
Main results	16	(*a*) Give unadjusted estimates and, if applicable, confounder-adjusted estimates and their precision (eg, 95% confidence interval). Make clear which confounders were adjusted for and why they were included	11-12
		(*b*) Report category boundaries when continuous variables were categorized	11-12
		(*c*) If relevant, consider translating estimates of relative risk into absolute risk for a meaningful time period	n/a
Other analyses	17	Report other analyses done—eg analyses of subgroups and interactions, and sensitivity analyses	n/a
**Discussion**			
Key results	18	Summarise key results with reference to study objectives	14
Limitations	19	Discuss limitations of the study, taking into account sources of potential bias or imprecision. Discuss both direction and magnitude of any potential bias	16-17
Interpretation	20	Give a cautious overall interpretation of results considering objectives, limitations, multiplicity of analyses, results from similar studies, and other relevant evidence	14-17
Generalisability	21	Discuss the generalisability (external validity) of the study results	16-17
**Other information**			
Funding	22	Give the source of funding and the role of the funders for the present study and, if applicable, for the original study on which the present article is based	18

For peer review only
